# Identification and validation of an inflammation-related lncRNAs signature for improving outcomes of patients in colorectal cancer

**DOI:** 10.3389/fgene.2022.955240

**Published:** 2022-09-30

**Authors:** Mengjia Huang, Yuqing Ye, Yi Chen, Junkai Zhu, Li Xu, Wenxuan Cheng, Xiaofan Lu, Fangrong Yan

**Affiliations:** State Key Laboratory of Natural Medicines, Research Center of Biostatistics and Computational Pharmacy, China Pharmaceutical University, Nanjing, China

**Keywords:** colorectal cancer, inflammation, lncRNAs, prognosis, immunotherapy

## Abstract

**Background:** Colorectal cancer is the fourth most deadly cancer worldwide. Although current treatment regimens have prolonged the survival of patients, the prognosis is still unsatisfactory. Inflammation and lncRNAs are closely related to tumor occurrence and development in CRC. Therefore, it is necessary to establish a new prognostic signature based on inflammation-related lncRNAs to improve the prognosis of patients with CRC.

**Methods:** LASSO-penalized Cox analysis was performed to construct a prognostic signature. Kaplan-Meier curves were used for survival analysis and ROC curves were used to measure the performance of the signature. Functional enrichment analysis was conducted to reveal the biological significance of the signature. The R package “maftool” and GISTIC2.0 algorithm were performed for analysis and visualization of genomic variations. The R package “pRRophetic”, CMap analysis and submap analysis were performed to predict response to chemotherapy and immunotherapy.

**Results:** An effective and independent prognostic signature, IRLncSig, was constructed based on sixteen inflammation-related lncRNAs. The IRLncSig was proved to be an independent prognostic indicator in CRC and was superior to clinical variables and the other four published signatures. The nomograms were constructed based on inflammation-related lncRNAs and detected by calibration curves. All samples were classified into two groups according to the median value, and we found frequent mutations of the TP53 gene in the high-risk group. We also found some significantly amplificated regions in the high-risk group, 8q24.3, 20q12, 8q22.3, and 20q13.2, which may regulate the inflammatory activity of cancer cells in CRC. Finally, we identified chemotherapeutic agents for high-risk patients and found that these patients were more likely to respond to immunotherapy, especially anti-CTLA4 therapy.

**Conclusion:** In short, we constructed a new signature based on sixteen inflammation-related lncRNAs to improve the outcomes of patients in CRC. Our findings have proved that the IRLncSig can be used as an effective and independent marker for predicting the survival of patients with CRC.

## Introduction

Colorectal cancer (CRC) is the fourth deadliest cancer worldwide, its incidence is closely related to lifestyle, heredity and environmental factors, and the incidence and death rate in women is approximately 25% lower than those in men (Dekker et al., 2019). CRC accounts for approximately 10% of cancer-related deaths every year (Bray et al., 2018). And it is prone to metastasis and invasion, especially in advanced patients (Li et al., 2020). CRC can be divided into rectal adenocarcinoma and colon adenocarcinoma based on anatomic location (Zuo et al., 2019). The treatment of colon cancer and rectal cancer is mainly surgical resection, and rectal surgery is more difficult than colon surgery. For advanced patients with CRC, oxaliplatin in combination with fluorouracil is the standard treatment (Xie et al., 2020). Although the current treatment strategies have prolonged the overall survival of patients, the 5-years survival rate of patients remains low, at just over 50% (Frampton and Houlston, 2016).

Long noncoding RNAs (lncRNAs) are involved in a great diversity of biological processes, and various mechanisms of lncRNA transcriptional regulation of gene expression have been demonstrated (Navarro et al., 2006; Rinn et al., 2007; Gupta et al., 2010). Increasing evidence suggests that the abnormal expression of lncRNAs is closely related to tumor progression. For example, MIR17HG, SNHG14 and VIM-AS1 can promote the tumorigenesis and metastasis of tumor cells in CRC (Rezanejad et al., 2018; Xu et al., 2019a; Wu et al., 2019). Furthermore, inflammation is a marker of cancer and can be caused by diverse factors, including infection, environmental, cell senescence and obesity (Terzi et al., 2010). Inflammation is double-sided in cancer: on the one hand, cytotoxic T lymphocytes, which can target specific cancer cells, and regulatory T cells, which can inhibit nonspecific inflammation, may contribute to the antitumor response (Long et al., 2017). On the other hand, inflammatory cells and the chemokines and cytokines they produce can affect the biological processes of cells in the tumor microenvironment, thus promoting tumorigenesis, development, malignant transformation, invasion and metastasis of tumors (Balkwill and Mantovani, 2001; Werb and Coussens, 2006; Grivennikov et al., 2010; Greten and Grivennikov, 2019). Sustained use of low-dose anti-inflammatory drugs can slow the tumor progression in the long term, which also illustrates the tumor-promoting role of inflammation in CRC (Friis et al., 2015). In addition, lncRNAs are closely related to immunity and inflammation and can regulate inflammation and immune-related signaling pathways through a variety of mechanisms (Xu et al., 2019b).

Here, we constructed a new prognostic signature, IRLncSig and demonstrated its stability and reliability in validation cohorts. Functional enrichment analysis, somatic mutation and copy number alterations analysis were performed to reveal the biological significance of the IRLncSig. Then, we identified potential therapeutic agents for high-risk patients and found that high-risk group may respond to immunotherapy, especially anti-CTLA4 therapy. Overall, the IRLncSig was a robust and independent marker for predicting the outcomes of patients, which provides a new basis for improving outcomes in CRC.

## Materials and methods

### Study cohorts and data preprocessing

The data used in this study mainly include three publicly available datasets, TCGA-COADREAD (including TCGA-COAD and TCGA-READ) for training and testing, GSE38832, GSE39582 and GSE72970 for validation. For the TCGA-COADREAD cohort, the expression profiles and clinical information, including age, sex, pathological stage, status, and overall survival (OS), were downloaded from TCGA (https://portal.gdc.cancer.gov/) using the R package “TCGAbiolinks”, and the expression value was converted into TPM values with log2 (TPM+1) for subsequent analysis. Samples lacking clinical information were excluded. In addition, the cohort was divided into the training set and testing set at a ratio of 7:3 using the R package “caret”. For the GSE39582, GSE38832 and GSE72970 cohorts, the expression profiles and clinical information were downloaded from the GEO database (https://www.ncbi.nlm.nih.gov/geo/). Similarly, samples lacking clinical information were removed. Detailed sample information of these datasets is summarized (Table 1).

**TABLE 1:** Summarized clinical characteristics of samples in this study.

	TCGA training	TCGA testing	GSE39582	GSE38832
**N**	414	177	572	122
**Age**				
>68	202 (48.8%)	75 (42.4%)	284 (49.7%)	-
≤68	212 (51.2%)	102 (57.6%)	288 (50.3%)	-
**Gender**				
Female	191 (46.1%)	81 (45.8%)	256 (44.8%)	-
Male	223 (53.9%)	96 (54.2%)	316 (55.2%)	-
**Stage**				
I	69 (16.7%)	32 (18.1%)	36 (6.3%)	18 (14.75)%
II	162 (39.1%)	53 (29.9%)	265 (46.3%)	35 (28.69%)
III	118 (28.5%)	54 (30.5%)	208 (36.4%)	39 (31.97%)
IV	51 (12.3%)	32 (18.1%)	59 (10.3%)	30 (24.59%)
unknown	14 (3.4%)	6 (3.4%)	4 (0.7%)	0 (0.00%)
**Survival**				
Dead	87 (21.0%)	35 (19.8%)	190 (33.2%)	28 (22.95%)
Alive	327 (79.0%)	142 (80.2%)	382 (66.8%)	94 (77.05%)

### Construction and validation of the IRLncSig

Genes associated with inflammation were downloaded from the gene database in NCBI (www.ncbi.nlm.nih.gov/gene) (Brown et al., 2015; Choe et al., 2021). First, we conducted Pearson correlation analysis to identify lncRNAs associated with inflammation (Pearson correlation coefficient: r > 0.3). Subsequently, univariate Cox regression analysis was performed to screen out lncRNAs with prognostic values (*p* < 0.01). To minimize the risk of overfitting, we used a multivariate Cox regression model with the least absolute shrinkage and selection operator (LASSO) with tenfold cross-validation to tune the optimal value of penalty parameter 𝜆 using the R package “glmnet”. Finally, a new signature, IRLncSig, was constructed based on sixteen inflammation-related lncRNAs. The IRLncSig was defined as follows:

$$IRLncSig=\overset{n}{\underset{i=1}{\sum }}{Coef}_{i}\times {Expr}_{i}$$

where ${Coef}_{i}$ is the coefficient of the lncRNA that was determined by the multivariate regression model, ${Expr}_{i}$ is the expression value of this lncRNA, and the IRLncSig is the computed risk score for each sample. We classified all samples into two groups according to the median value and we used the Kaplan-Meier method for survival analysis and the Log-rank test to detect differences using the R package “survival” (Hazra and Gogtay, 2016a). In addition, the receiver operating characteristic (ROC) curves of the IRLncSig for 1-year, 3-years and 5-years survival were plotted using the R package “survivalROC”.

### Construction and evaluation of nomograms and calibration curves

A nomogram model was constructed according to 16 inflammation-related lncRNAs to predict 1-,3-, and 5-years OS using the R package “rms”. Then, we use the calibration curves of 1-,3-, and 5-years to verify the performance of nomogram models by bootstrap method with 1,000 resamples.

### Independence of prognostic effect of the IRLncSig

To evaluate the independence of the IRLncSig, we conducted multivariate regression analysis on age, sex, pathologic stage and the IRLncSig. We also performed stratification analysis by pathologic stage and age respectively. For the pathological stage, we divided samples into two groups, “stage I/II” and “stage III/IV” after removing samples with missing values. For age, we classified samples into two groups based on the median value. Kaplan–Meier curves and Log-rank test were used for evaluating the differences in OS using the R package “survival”.

### Biological function and pathway analysis

To further understand the biological significance of the IRLncSig, we performed gene set enrichment analysis (GSEA) through the R package “clusterProfiler” (Yu et al., 2012). The functional annotation gene set (msigdb.v7.2. symbols.gmt) was downloaded from the MSigDB database (http://www.gsea-msigdb.org/gsea/msigdb). First, we obtained differentially expressed genes using the R package “limma” and sorted these genes by log2FoldChange (Ritchie et al., 2015). Then, we conducted GSEA based on the pre-ranked gene list (Subramanian et al., 2005). In addition, ten oncogenic pathways and their gene signatures were obtained from published literature (Sanchez-Vega et al., 2018). Gene set variation analysis (GSVA) was used to calculate the enrichment score of carcinogenic pathways.

### Somatic mutation and copy number variation analysis

Somatic mutation data were obtained from cBioPortal (https://www.cbioportal.org/) and the R package “maftools” was used to analyze and visualize (Mayakonda et al., 2018). And the MutSigCV method was performed to identify driver genes in CRC (Lawrence et al., 2013).

Copy number variation data were obtained from Fire Browse (http://firebrowse.org/) and the Genomic Identification of Significant Targets in Cancer (GISTIC2.0, GenePattern) algorithm was performed to analyze and compare genomic alterations between two risk groups, including gains and losses (Mermel et al., 2011). The confidence level was set to 0.95 (Yang et al., 2020a; Luo et al., 2020).

### Drug sensitivity analysis

We predicted the sensitivity of each patient to chemotherapeutic agents by the R package “pRRophetic” (Paul et al., 2014). All 138 chemotherapy drugs were included in this package. The estimated IC50 value for each patient treated with a specific chemotherapy drug was obtained through the function “pRRopheticPredict”. Then, we screened out drugs with higher sensitivity for high-risk group patients. In addition, we used CMap analysis (https://clue.io) to determine agents, with which gene expression value increased in high-risk patients but decreased by treatment (scores <0) (Chen et al., 2020). Finally, we set the intersection of agents obtained in the two steps to determine the final potential treatment agents for patients with high risk.

### Characterization of immune microenvironment and prediction of immunotherapy response

To further reveal the differences in immune microenvironment between the two groups, we first estimated the abundance of 22 immune cells and 2 stromal cells using single sample gene set enrichment analysis (ssGSEA). We also obtained 10 immune checkpoints from published literature and compared the difference in expression between the two groups (Yang et al., 2020b). In addition, the submap algorithm was used to evaluate the response of patients to immunotherapy based on the dataset of 47 patients with cutaneous melanoma (Hoshida et al., 2007). The method was performed by the Submap module in GenePattern.

### Performance comparison of the IRLncSig with other clinical variables and existing prognostic signatures

ROC curves were used to estimate the 1-year, 3-years and 5-years survival. The clinical variables age and pathological stage were considered. In detail, the IRLncSig and age were treated as continuous variables, while the pathologic stage was coded as binary, “Stage I/II” and “Stage III/IV”. We also compared the IRLncSig with four published prognostic signatures in CRC: a 15-gene signature derived from Yang’s study (Yang et al., 2021), a 6-lncRNA signature derived from Li’s study (Li et al., 2020), a 6-gene signature derived from Dai’s study (Dai et al., 2020), and a 7-gene signature derived from Lu’s study (Lu et al., 2021).

### Statistical analysis

All statistical analyses were conducted by R version 4.0.2. Statistical test methods used in this study mainly include the Wilcoxon test for two groups, the Kruskal-Wallis test for more than two groups, and the Chi-Square test or Fisher’s exact test for contingency table variables (Hazra and Gogtay, 2016b). All statistical tests were two-sided, and it was considered statistically significant with *p* < 0.05.

## Results

### Construction and validation of the IRLncSig based on inflammation-related lncRNAs

**FIGURE 1:**
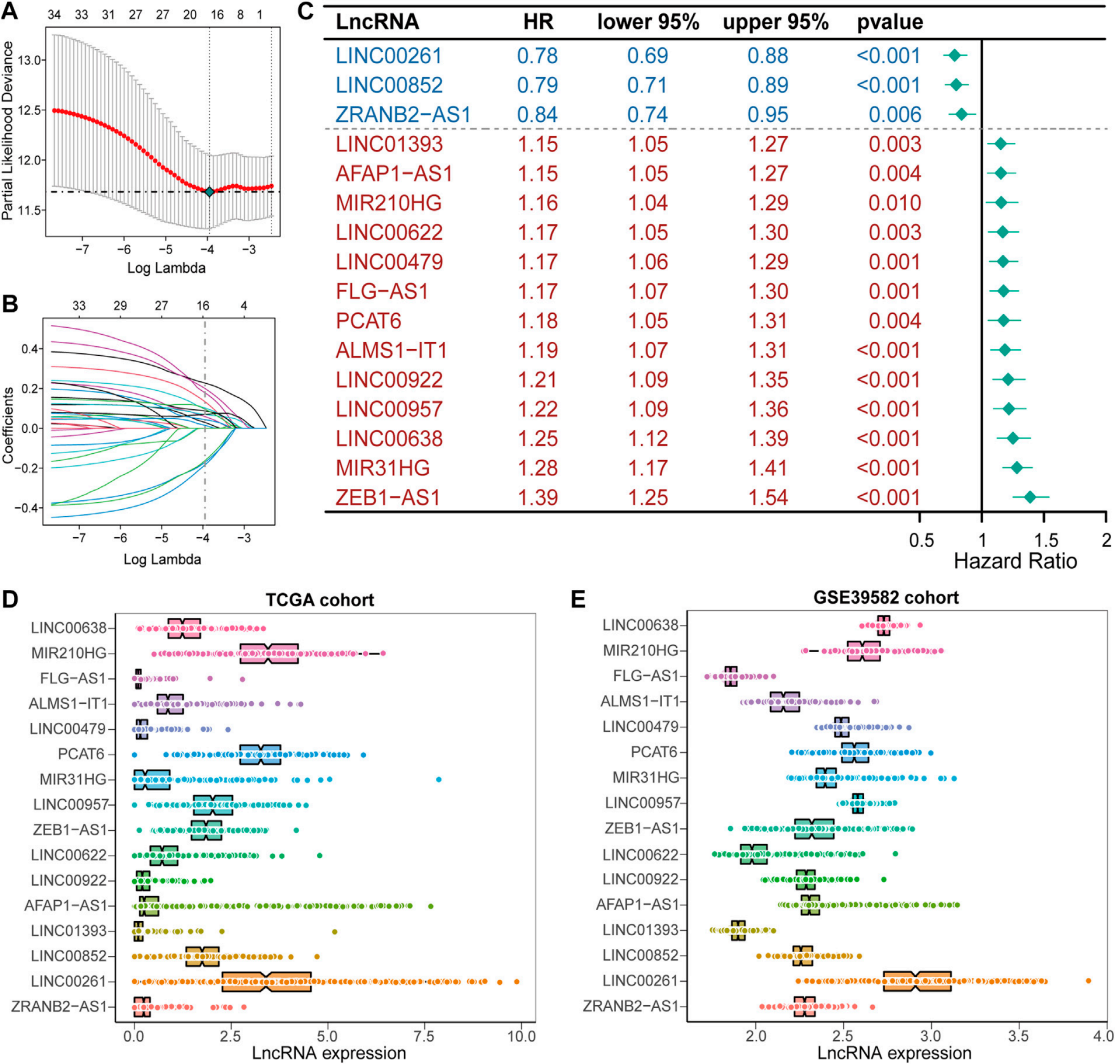
Construction of the prognostic signature, IRLncSig **(A)** Feature selection using the LASSO-penalized regression by 10-fold cross-validation with minimum criteria. **(B)** LASSO coefficient profiles of the 34 inflammation-related lncRNAs with nonzero coefficients **(C)** Forest plot for identified sixteen lncRNAs. **(D,E)** Boxviolin plot for the expression level of the sixteen lncRNAs.

A detailed workflow for prognostic signature construction and analysis was developed (Supplementary Figure S1). We downloaded 2,826 inflammation-related genes from NCBI and finally obtained 2,380 inflammation-related genes after the intersection with expression profile data. We screened out 934 lncRNAs strongly correlated with inflammatory genes (Pearson correlation coefficient: r > 0.3) and 34 lncRNAs with prognostic values (*p* < 0.01; Supplementary Figure S2). Next, sixteen lncRNAs with prognostic values were identified by univariate and LASSO-penalized regression analysis (Table 2; Figures 1A,B). The sixteen inflammation-related lncRNAs, which were independently correlated with survival, were preserved as prognostic factors. Of them, thirteen lncRNAs were related to worse prognosis and presented as “risk” factors, while the remaining three lncRNAs were related to better prognosis and presented as “protective” factors (Figure 1C).Figures 1D,E show the expression levels of the sixteen lncRNAs in the TCGA and GSE39582 datasets, respectively.

**TABLE 2:** Sixteen inflammation-related lncRNAs with prognostic value.

Ensemble id	Gene name	Coefficient	HR	*p*.value
ENSG00000235079	ZRANB2-AS1	−0.18	0.84 (0.74–0.95)	0.006
ENSG00000259974	LINC00261	−0.17	0.78 (0.69–0.88)	<0.001
ENSG00000231177	LINC00852	−0.16	0.79 (0.71–0.89)	<0.001
ENSG00000225535	LINC01393	0.01	1.15 (1.05–1.27)	0.003
ENSG00000272620	AFAP1-AS1	0.03	1.15 (1.05–1.27)	0.004
ENSG00000261742	LINC00922	0.04	1.21 (1.09–1.35)	<0.001
ENSG00000260941	LINC00622	0.06	1.17 (1.05–1.30)	0.003
ENSG00000237036	ZEB1-AS1	0.07	1.39 (1.25–1.54)	<0.001
ENSG00000235314	LINC00957	0.07	1.22 (1.09–1.36)	<0.001
ENSG00000171889	MIR31HG	0.09	1.28 (1.17–1.41)	<0.001
ENSG00000228288	PCAT6	0.10	1.18 (1.05–1.31)	0.004
ENSG00000236384	LINC00479	0.10	1.17 (1.06–1.29)	0.001
ENSG00000230002	ALMS1-IT1	0.13	1.19 (1.07–1.31)	<0.001
ENSG00000237975	FLG-AS1	0.18	1.17 (1.07–1.30)	0.001
ENSG00000247095	MIR210HG	0.20	1.16 (1.04–1.29)	0.010
ENSG00000258701	LINC00638	0.23	1.25 (1.12–1.39)	<0.001

**FIGURE 2:**
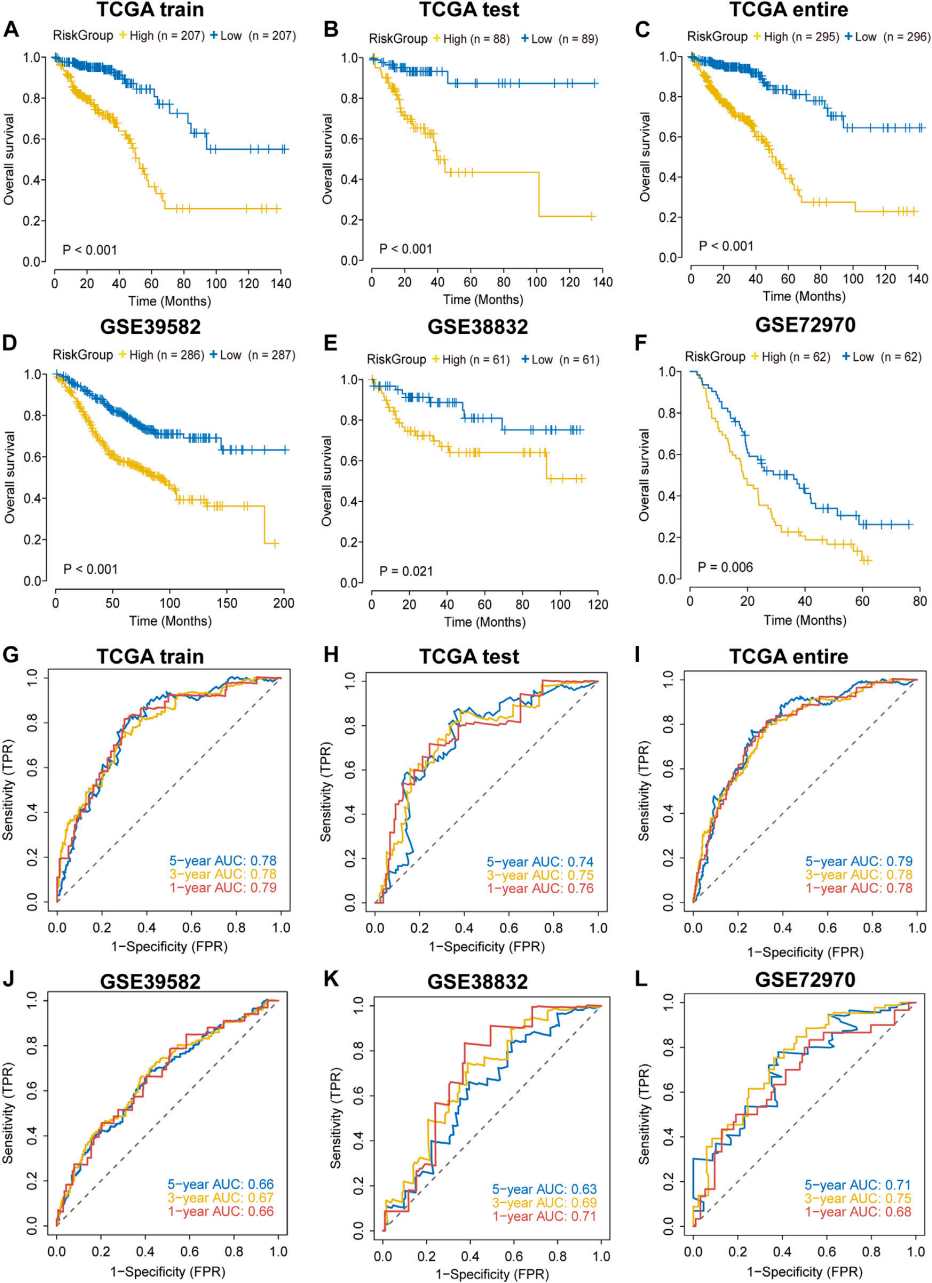
Validation of the performance of the IRLncSig for prognostic prediction **(A–F)** Survival analysis for two groups. The *p* values were calculated by the Log-rank test. **(G–L)** ROC curves for predicting 1-, 3- and 5-years OS.

In each cohort, we calculated the risk score according to the coefficients and expression values of the sixteen lncRNAs and divided samples into high-risk and low-risk groups based on the median value. Survival analysis indicated that high-risk group had a worse prognosis in the TCGA training cohort (Figure 2A). We found similar results in TCGA testing, TCGA entire, GSE38832, GSE39582 and GSE72970 datasets (Figures 2B–F). The AUC value was 0.79 in the first year and 0.78 both in the third and fifth year in the TCGA training dataset (Figure 2G), and the AUC values in other validation datasets are shown in Figures 2H–L. In addition, we found that the mortality was elevated with the increase of the risk score (Supplementary Figure S3). The “risk” lncRNAs were highly expressed in high-risk group and the protective lncRNAs were highly expressed in the low-risk group (Supplementary Figure S3). These results indicated that the IRLncSig could act as an effective prognostic marker in CRC.

### Construction and evaluation of nomograms and calibration curves

**FIGURE 3:**
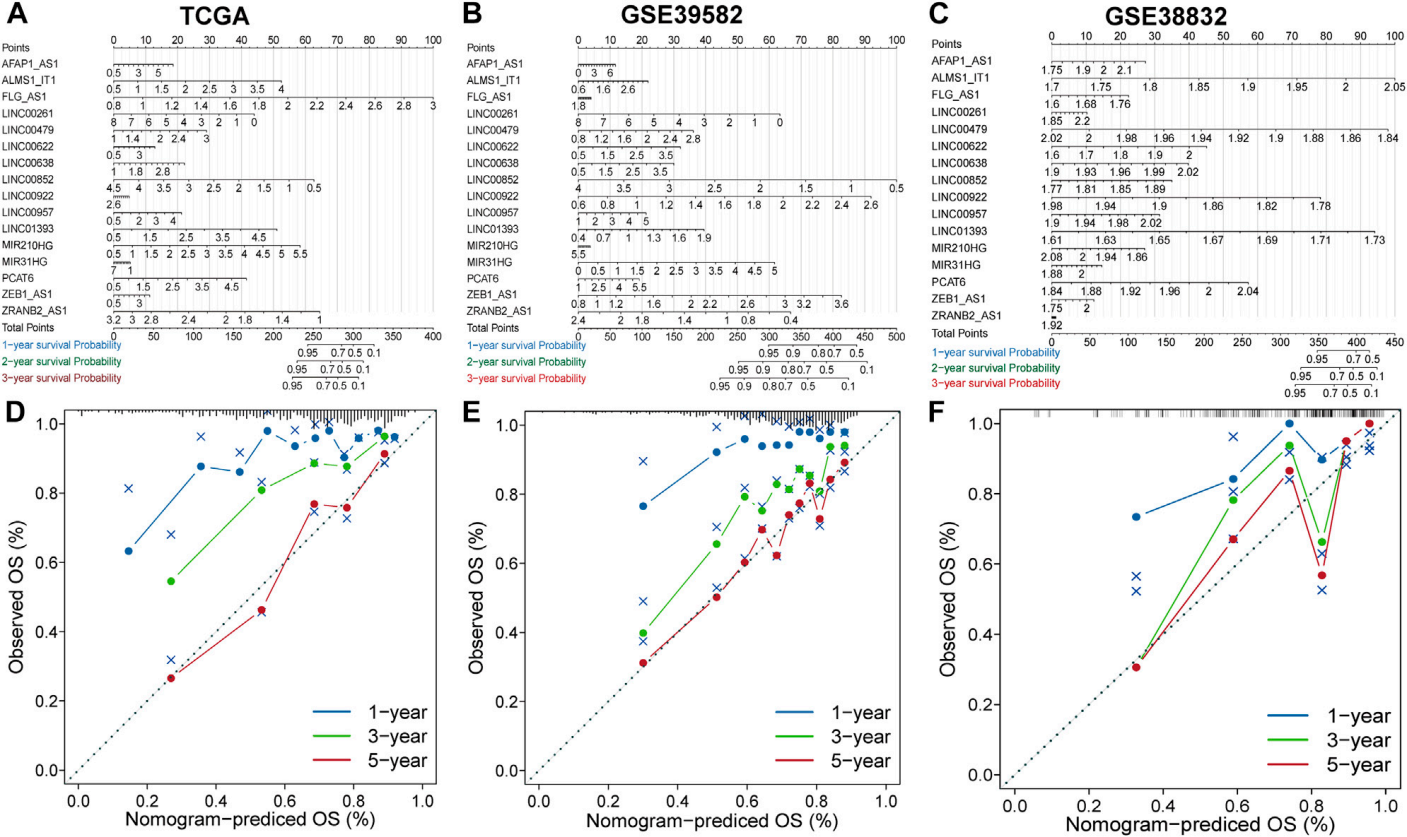
Construction and evaluation of prognostic nomograms and calibration curves **(A–C)** The nomogram predicts the probability of 1-, 3-, and 5-years OS. **(D–F)** The calibration plot of the nomogram predicts the probability of 1-, 3-, and 5-years OS.

The nomogram comprising the sixteen lncRNAs was fabricated to estimate the 1-year, 3-years and 5-years OS in the TCGA dataset (Figure 3A). The calibration curve for the observed versus predicted probability of the 1-year, 3-years and 5-years OS revealed consistency (Figure 3D). Similar results could be observed in GSE39582 and GSE38832 validation cohorts Figures 3B,C; Figures 3E,F). These results suggested that the nomogram model based on the sixteen lncRNAs could help predict the prognosis of patients in CRC.

### Independence of prognostic effects of the IRLncSig

**FIGURE 4:**
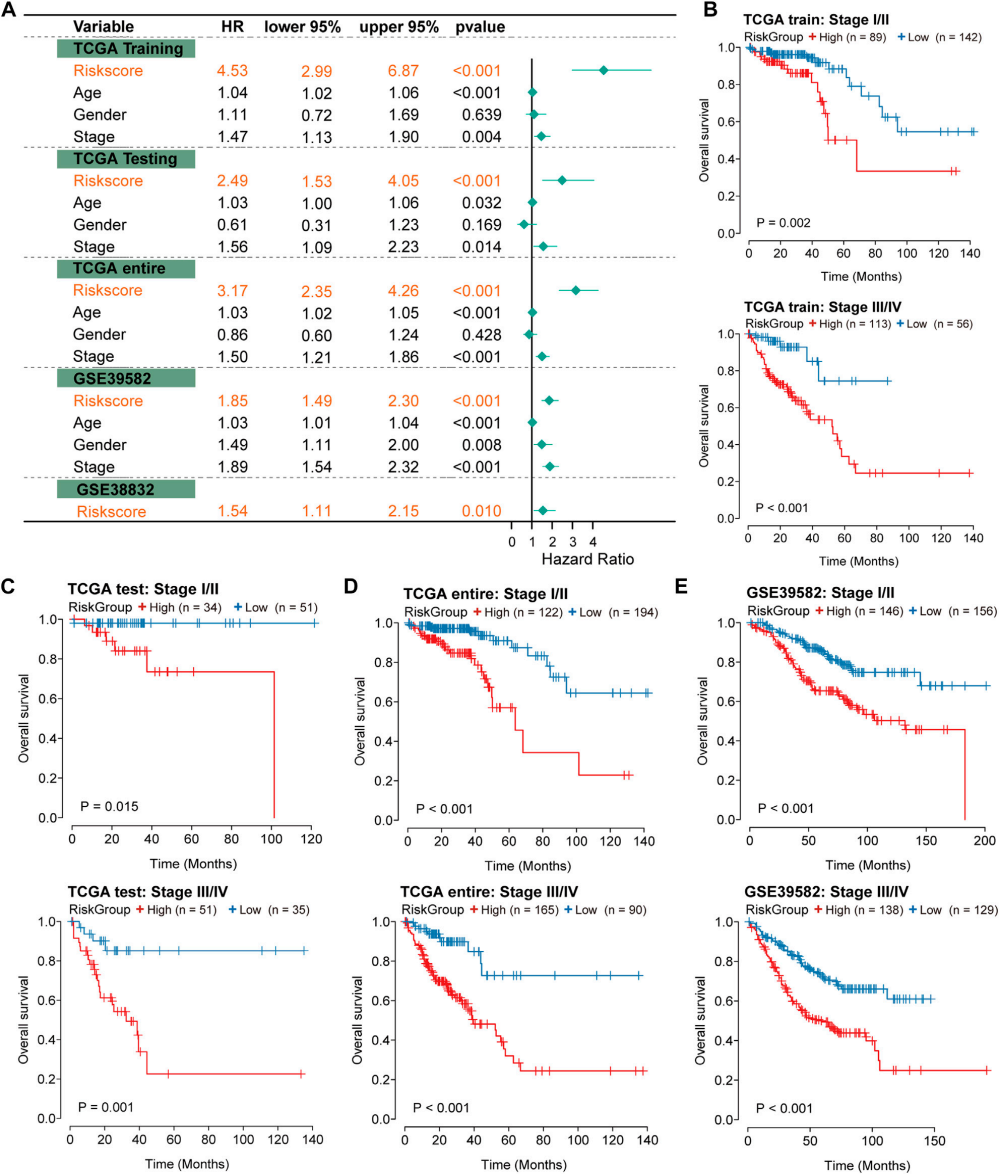
Independence of prognostic effect of the IRLncSig **(A)** Forest plot for the IRLncSig and other clinical factors by multivariate regression analysis. **(B–E)** Stratification analysis by pathological stage. The *p* values were calculated by the Log-rank test.

To evaluate the independence of the IRLncSig, we conducted multivariate regression analysis on age, sex, pathologic stage and the IRLncSig. The results showed that the IRLncSig could act as an independent prognostic signature in all cohorts (Figure 4A). In addition to the IRLncSig, the pathologic stage and age were observed to be highly significant (Figure 4A). Therefore, we performed stratification analysis by pathologic stage and age respectively. For the pathological stage, the results indicated that there were significant differences in OS between two groups “stage I/II” and “stage III/IV” in all datasets Figures 4B–E). Similar results were found in the stratification analysis of age (Supplementary Figure S4). These results all indicated that the IRLncSig could act as an independent and effective marker in CRC.

### Biological function and pathway analysis

**FIGURE 5:**
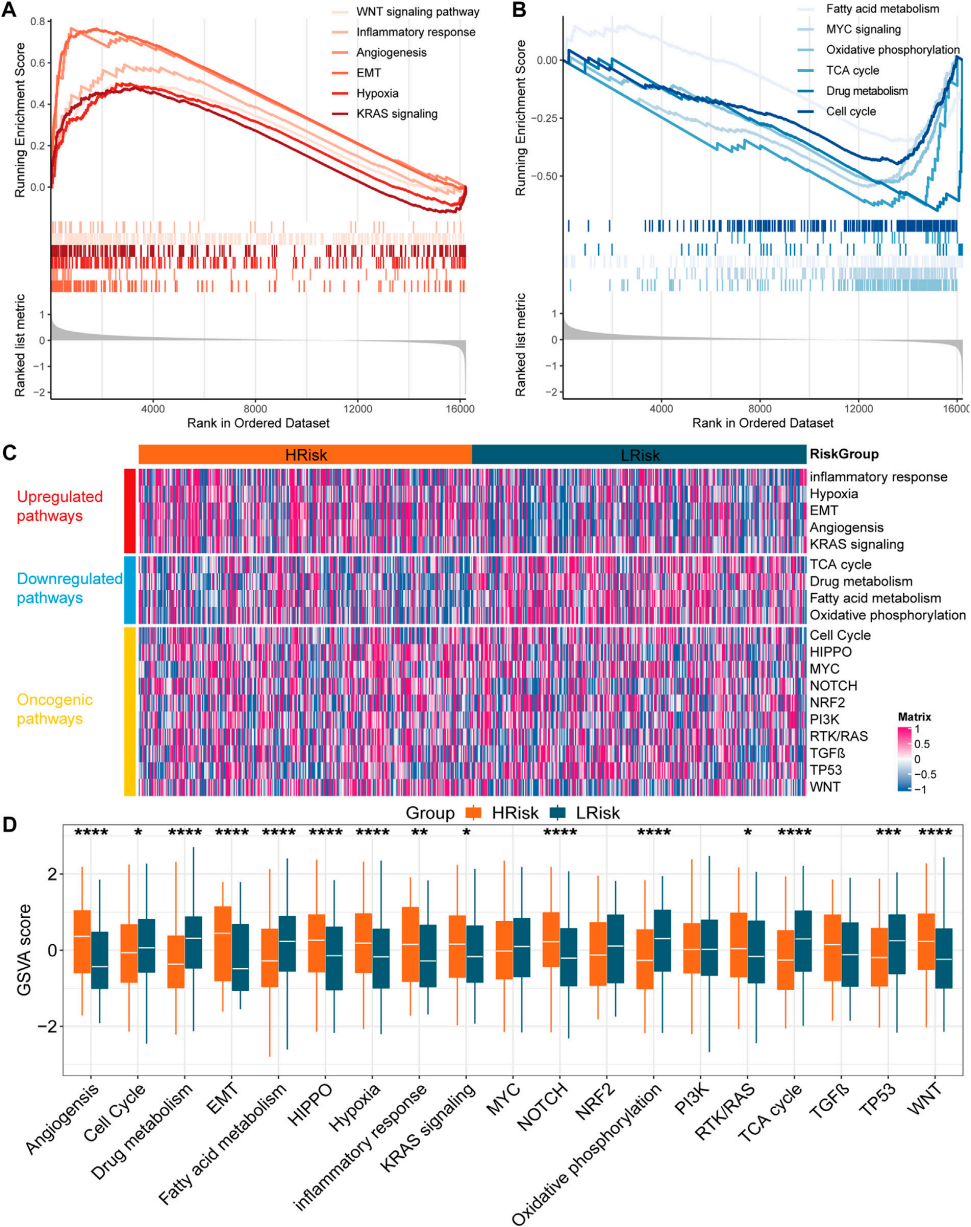
Function enrichment analysis in the TCGA cohort **(A,B)** Gene set enrichment analysis based on GO, KEGG, Hallmark and Reactome. **(C)** Heatmap for the GSVA score of upregulated, downregulated and oncogenic pathways **(D)** Boxplot for the GSVA score of upregulated, downregulated and oncogenic pathways between two subgroups. The *p* values were calculated by Wilcoxon test (*p < 0.05; **p < 0.01; ***p < 0.001; ****p < 0.0,001).

To further understand the biological significance of the IRLncSig, differential expression analysis was first performed. Next, GSEA was performed based on Gene Ontology (GO), Kyoto Encyclopedia of Genes and Genomes (KEGG), Hallmark and Reactome gene sets. The results revealed that epithelial-mesenchymal transition (EMT), angiogenesis, inflammatory response, hypoxia, WNT and KRAS signaling pathways were enriched in the high-risk group (Figure 5A). Oxidative phosphorylation, MYC signaling, cell cycle, fatty acid metabolism, drug metabolism and TCA cycle were enriched in the low-risk group (Figure 5B). Next, GSVA was used to estimate the abundance of up-regulated, down-regulated, and carcinogenic pathways. We found that the up-regulated pathways exhibited a higher GSVA score in the high-risk group, while the down-regulated pathways exhibited the opposite (Figures 5C,D). In addition, HIPPO, NOTCH, RTK/RAS and WNT carcinogenic pathways had higher GSVA score in the high-risk group, while cell cycle and TP53 signaling had higher GSVA score in the low-risk group (Figures 5C,D). The results were similar in the GSE39582 validation set (Supplementary Figure S5).

### Characterization of somatic mutation and copy number variations

We first identified driver genes in CRC by MutSigCV (q < 0.05; Supplementary Figure S6A). Then, we found that the mutation rate of gene TP53 was significantly higher in the high-risk group (Supplementary Figure S6B). To further verify the prognostic independence of the IRLncSig, we divided samples into four subgroups based on TP53 mutation status and risk group: Mutated in HRisk, Mutated in LRisk, Wild in HRisk and Wild in LRisk. Survival analysis showed that the OS of both mutated-type and wild-type in the high-risk group was significantly lower (Supplementary Figure S6C). These results again demonstrated the independence of the IRLncSig in predicting outcomes of patients in CRC.

**FIGURE 6:**
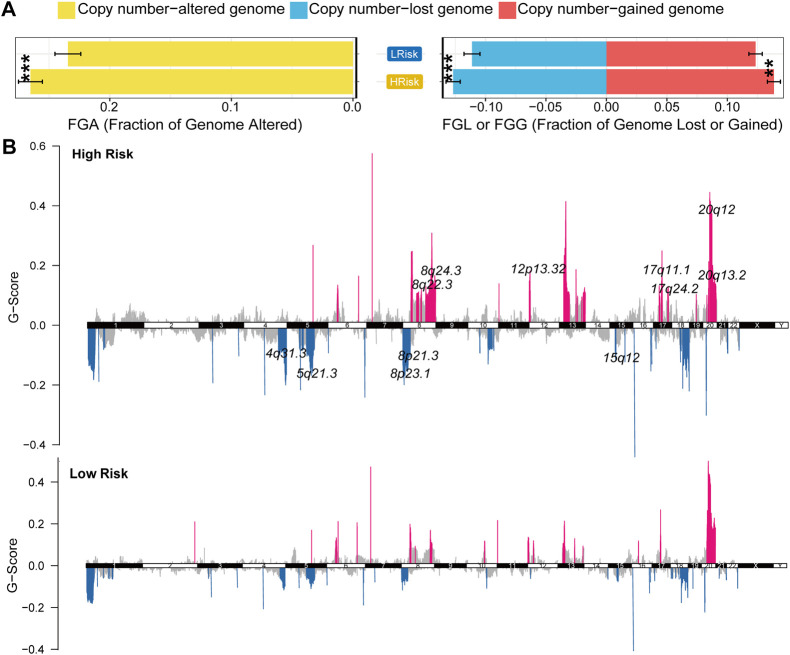
Characterization of copy number variation in the entire TCGA cohort **(A)** The difference in genomic alterations between the two groups. The *p* values were calculated by Wilcoxon test (*p < 0.05; **p < 0.01; ***p < 0.001; ****p < 0.0,001) **(B)** GISTIC cytoband of copy number alteration for all samples. (*p < 0.05; **p < 0.01; ***p < 0.001; ****p < 0.0,001).

GISTIC2.0 was used to analyze the copy number variation (Mermel et al., 2011). First, we compared genome alterations between two groups, including the fraction of genome altered (FGA), the fraction of genome lost (FGL) and the fraction of genome gained (FGG). Results showed that high-risk group exhibited more genome alterations, including FGA, FGL, and FGG (Figure 6A). We also found seven significantly amplificated regions (8q22.3, 8q24.3, 12p13.32, 17q11.1, 17q24.2, 20q12, 20q13.2) and five significantly deleted regions (4q31.3, 5q21.3, 8p21.3, 8p23.1, 15q12) in the high-risk group (Figure 5B). We further analyzed the correlation between the copy number of these amplified genes and their expression values and identified eleven inflammatory genes in the regions 8q24.3, 20q12, 8q22.3 and 20q13.2 with a strongly positive correlation (Table 3; Supplementary Figure S6D). These genes were highly expressed in the high-risk group except for the genes PLCG1, NFATC2, TOP1 and CYP24A1 (Supplementary Figure S6E). These results suggested that the four regions may regulate the inflammatory activity of cancer cells, further clarifying that alterations in specific chromosomal regions may influence the heterogeneity of CRC.

**TABLE 3:** Eleven inflammatory genes with strong correlation.

GenesSymbol	Spearman'r	*p*.value	Cytoband
HSF1	0.695	4.85E-85	8q24.3
SHARPIN	0.661	2.23E-74	8q24.3
PLCG1	0.653	6.62E-72	20q12
PTK2	0.638	8.56E-68	8q24.3
ZNF7	0.632	4.98E-66	8q24.3
YWHAZ	0.526	1.06E-42	8q22.3
SCRIB	0.522	6.42E-42	8q24.3
NFATC2	0.486	1.07E-35	20q13.2
TOP1	0.412	2.86E-25	20q12
GSDMD	0.401	7.54E-24	8q24.3
CYP24A1	0.304	6.92E-14	20q13.2

### Identification of potential therapeutic agents for patients in CRC

**FIGURE 7:**
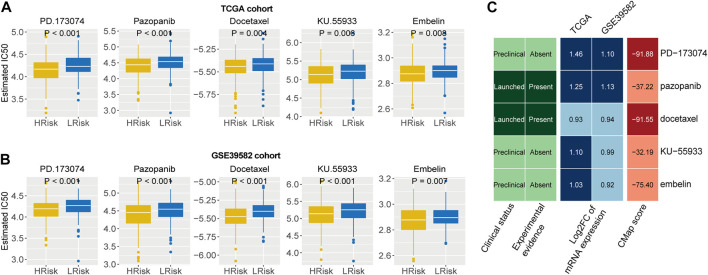
Identification of potential therapeutic agents for high-risk patients **(A,B)** Five agents with higher drug sensitivity in the high-risk group. The *p* values were calculated by the Wilcoxon test. **(C)** CMap analysis of five drugs with potential therapeutic agents for high risk patients.

We first identified five drugs with higher sensitivity for high-risk group patients from 138 chemotherapy drugs Figures 7A,B). Then, the correlation of IC50 values and target expression level with the IRLncSig for five drugs was analyzed (Supplementary Figure S7). We further tested the efficacy of these drugs by CMap analysis based on differentially expressed genes. The results showed that PD.173,074 (CMap score = −91.88) and Docetaxel (CMap score = −91.55) might be potential therapeutic drugs for patients with high risk (Figure 7C). PD-173074 is a fibroblast growth factor receptor (FGFR) inhibitor. FGFR1 is overexpressed and is considered a therapeutic target in CRC (Jang, 2005; Göke et al., 2013). Studies have also shown that its overexpression is associated with liver metastasis of CRC (Kwak et al., 2015). Docetaxel is a tubulin polymerization inhibitor, and its target is BCL-2, which is an oncogene and can inhibit apoptosis.

### Characterization of immune microenvironment between two risk groups

We first characterized the cellular interactions in the immune microenvironment, including 22 immune cells and 2 stromal cells, and the results showed that there was a strong positive correlation between these cells (Supplementary Figure S8A). CD8 T cells (HR: 0.84, 95% CI: 0.72–0.99, *p* = 0.038) and activated memory CD4 T cells (HR: 0.80, 95% CI: 0.67–0.95, *p* = 0.013) exhibited prognostic value in CRC (Supplementary Figure S8B). For activated memory CD4 T cells, the group with higher immune infiltration had a better prognosis (*p* = 0.004; Supplementary Figure S8C). However, there was no significant difference for CD8 T cells (*p* = 0.125; Supplementary Figure S8D). We also analyzed the correlation between immune cell infiltration and inflammation-related lncRNAs (Supplementary Figure S8E).

**FIGURE 8:**
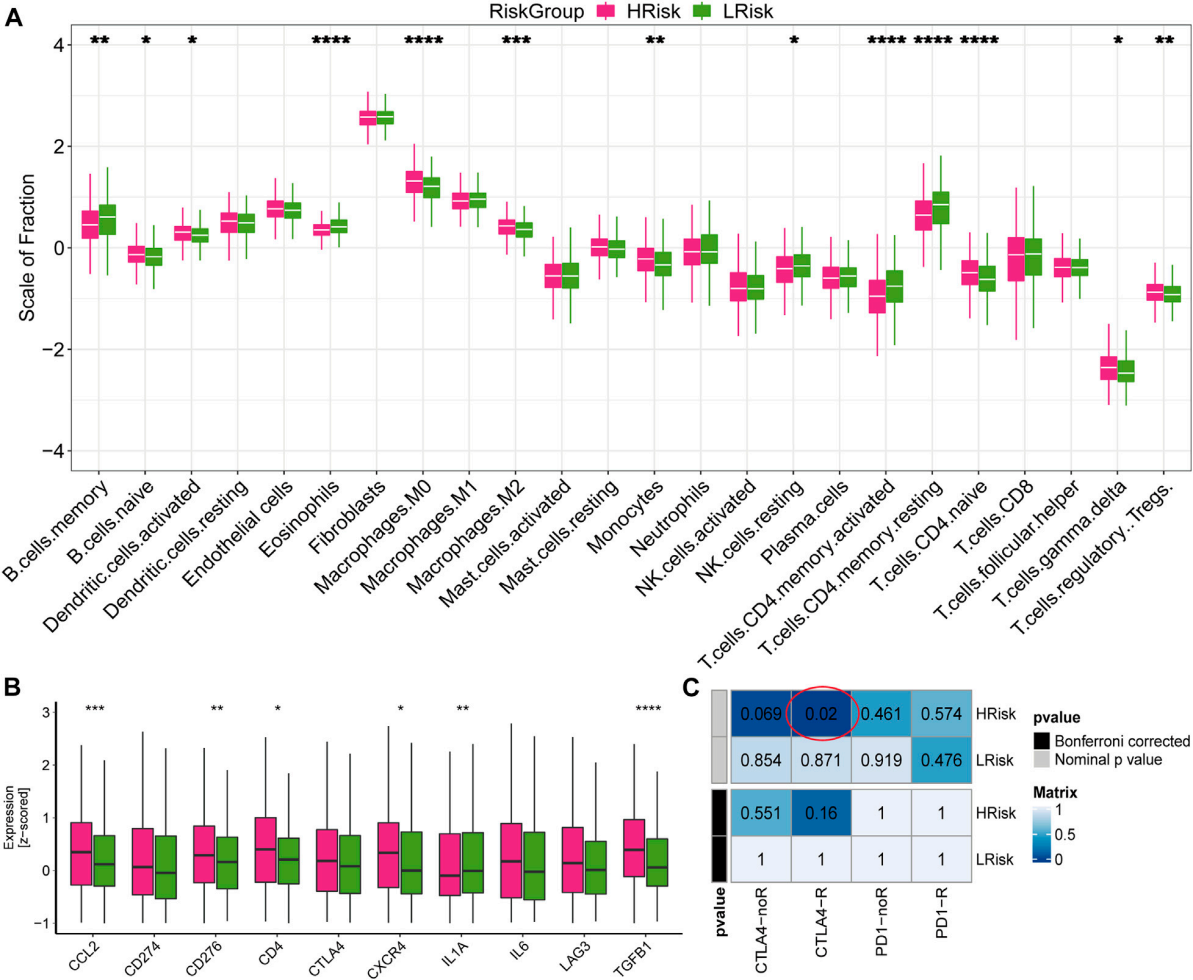
Immune infiltration and the response to immunotherapy in TCGA cohort **(A)** Boxplot for the abundance of 22 immune cells and 2 stromal cells estimated by ssGSEA. The *p* values were calculated by Wilcoxon test (*p < 0.05; **p < 0.01; ***p < 0.001; ****p < 0.0,001) **(B)** Boxplot for the expression level of 10 immune checkpoints. The *p* values were calculated by Wilcoxon test (*p < 0.05; **p < 0.01; ***p < 0.001; ****p < 0.0,001). **(C)** Submap analysis for predicting the response to immunotherapy.

To further explore the response to immunotherapy of patients, we analyzed the infiltration levels of immune cells and stromal cells in the immune microenvironment. We found that the abundance of naive B cells, activated dendritic cells, macrophages M0, macrophages M2, monocytes, naive CD4 T cells, regulatory T cells and gamma delta T cells were higher in the high-risk group, while the abundance of memory B cells, eosinophils, NK cells, activated memory CD4 T cells and resting memory CD4 T cells were higher in the low-risk group (Figure 8A). We also found that high-risk group exhibited higher expression values of immune checkpoints, including CCL2, CD276, CD4, CXCR4, IL1A, and TGFB1 (Figure 8B). In addition, the submap analysis showed that high-risk group was more likely to respond to anti-CTLA-4 immunotherapy (nominal *p* = 0.02; Figure 8C). However, no difference was found in anti-PD1 immunotherapy, which may need further study. These results were verified in the GSE39582 cohort (Supplementary Figure S9). Collectively, high-risk group exhibited higher immune cell infiltration and expression of immune checkpoints, suggesting that patients in the high-risk group may be responsive to immunotherapy, especially anti-CTLA4 therapy.

### Performance comparison of the IRLncSig with other clinical variables and existing prognostic signatures

**FIGURE 9:**
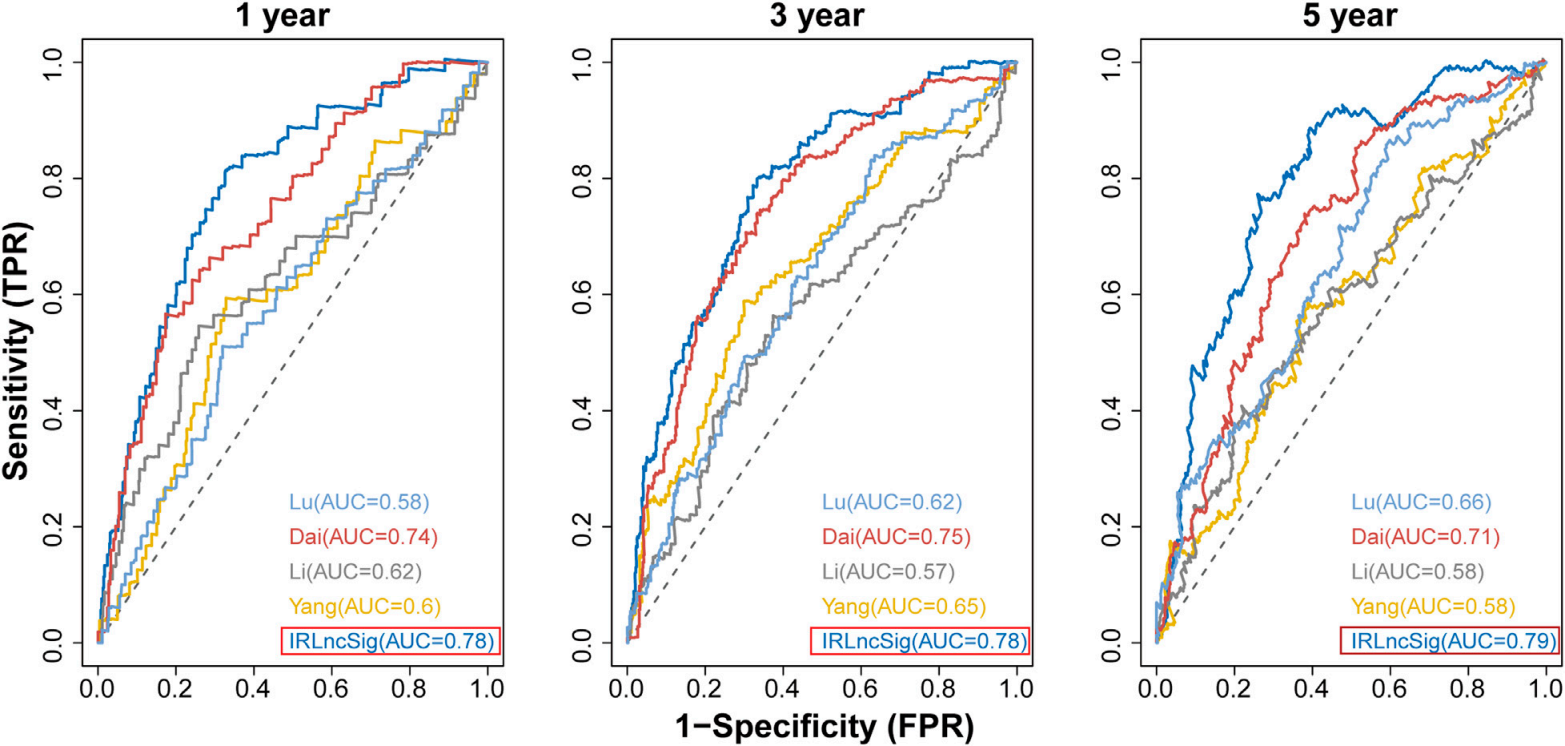
Comparison of IRLinSig with other existing prognostic signatures.

We compared the IRLncSig with four recently published prognostic signatures in CRC: a 15-gene signature derived from Yang’s study (Yang et al., 2021), a 6-lncRNA signature derived from Li’s study (Li et al., 2020), a 6-gene signature derived from Dai’s study (Dai et al., 2020), and a 7-gene signature derived from Lu’s study (Lu et al., 2021). The results showed that the AUC values at 1-year, 3-years and 5-years were 0.78, 0.78 and 0.79, respectively, which were all higher than those of the other four signatures (Figure 9). The results proved that the IRLncSig had better predictive performance than the other four published signatures in CRC. In addition, the performance of the signature was better than that of the clinical variables, age and pathologic stage (Supplementary Figure S10).

Finally, we associated the IRLncSig with the CMS subtypes established by previous studies (Guinney et al., 2015). The CMS4 subtypes had the worst prognosis and the highest risk score (Supplementary Figures S11A,B), and high-risk group exhibited a higher proportion of CMS4 subtype (*p* < 0.001; Supplementary Figure S11C). We also applied the signature to pan-cancer and found that it showed prognostic value in liver hepatocellular carcinoma (LIHC; HR: 1.47, 95% CI: 1.22–1.76, *p* < 0.001), uterine corpus endometrial carcinoma (UCEC; HR: 1.38, 95% CI: 1.14–1.67, *p* < 0.001), kidney renal clear cell carcinoma (KIRC; HR: 1.25, 95% CI: 1.11–1.41, *p* < 0.001), head and neck squamous cell carcinoma (HNSC; HR: 1.20, 95% CI: 1.05–1.36, *p* = 0.006), brain lower grade glioma (LGG; HR: 0.81, 95% CI: 0.68–0.97, *p* = 0.020), uveal melanoma (UVM; HR: 0.59, 95% CI: 0.40–0.89, *p* = 0.012) (Supplementary Figure S12).

## Discussion

CRC is a common cancer of the digestive tract, with morbidity and mortality among the top five in the world (Kuipers et al., 2015; Qaderi et al., 2020). More than 1.4 million people have been diagnosed as CRC, and more than 0.5 million people have died of this tumor in a year (Jeffery et al., 2007; Siegel et al., 2020). Approximately 30% of patients have tumor recurrence within 3 years after surgery and adjuvant chemotherapy (Sargent et al., 2009). Many studies have shown that the construction of prognostic signatures could accelerate the prognostic assessment of patients in CRC (Huang et al., 2020). In this study, we constructed a robust prognostic signature, IRLncSig, based on sixteen inflammation-related lncRNAs to improve the prognosis of patients with CRC. The predictive performance of the IRLncSig was verified on TCGA, GSE38832, GSE39582, and GSE72970 cohorts. Then, samples were divided into two risk groups based on the median value in each dataset and Kaplan–Meier curves exhibited that patients in the low-risk group had a better prognosis. The performance of the IRLncSig was also proved to outperform traditional clinical predictors and four published signatures in CRC. Subgroup analyses showed that the signature could predict outcomes in different subgroups of patients. These results indicated that the IRLncSig was an independent and effective marker in predicting the prognosis of patients with CRC.

To better understand the biological significance of the IRLncSig, we conducted enrichment analysis and found that high-risk group mainly enriched in EMT, angiogenesis, inflammatory response, hypoxia, WNT signaling and KRAS signaling pathways. EMT can promote tumor progression, including CRC, especially when it involves invasion and metastasis (Arias, 2001; Bates and Mercurio, 2005). Angiogenesis is a hallmark of cancer and it is associated with tumor progression and metastasis in some cancers, such as CRC, skin melanoma, breast cancer, prostate cancer and lung cancer (Yuji et al., 1996). Moreover, carcinogenic pathways, including HIPPO, NOTCH and RTK/RAS were enriched in the high-risk group. Low-risk group enriched mainly in oxidative phosphorylation, MYC signaling, cell cycle, fatty acid metabolism, drug metabolism and TCA cycle. Restoration of oxidative phosphorylation levels is one of the characteristics of tumor development and progression (Maiuri and Kroemer, 2015). The tricarboxylic acid cycle, also known as the citric acid cycle, is a common metabolic pathway in the human body. It is the hub of the metabolic links between the three nutrients, including carbohydrates, lipids and amino acids. The dysfunction of TCA is one of the causes of human disease and tumor formation (Brière et al., 2007). Several studies have shown that inflammation has been implicated in EMT (Suarez-Carmona et al., 2017), angiogenesis (Ping, 2012; Aguilar-Cazares et al., 2019), hypoxia (Mamlouk and Wielockx, 2013), WNT signaling (Yang et al., 2008), KRAS signaling (Kitajima et al., 2016), MYC signaling (Sipos et al., 2016) and lipid metabolism (Zuo et al., 2020). Therefore, it is speculated that the IRLncSig constructed based on inflammation-related lncRNAs may impact the activity of these pathways through inflammatory responses.

Gene mutations are implicated in cancer. Among them, TP53 is an important tumor suppressor gene and the wild-type causes apoptosis of cancer cells preventing carcinogenesis, and TP53 mutation will increase the probability of carcinogenesis (Guimaraes and Hainaut, 2002). Pan-cancer studies have shown that TP53 mutations have been found in a variety of malignancies and the mutation rate is 43.2% in CRC (Olivier et al., 2010). A large number of studies have shown that TP53 mutation is related to poor survival in CRC (Iacopetta, 2003). Here, we found that the TP53 gene was frequently mutated in the high-risk group. We also found that the prognostic effect of the IRLncSig was independent of TP53 mutation status, which again demonstrated the independence of the IRLncSig in predicting outcomes of patients in CRC.

Copy number variation is an important component of genome alterations, which affects gene expression and the activity of signaling pathways. Here, we found that high-risk group had more genome alterations. We also found seven significantly amplificated regions in the high-risk group, 8q22.3, 8q24.3, 12p13.32, 17q11.1, 17q24.2, 20q12, and 20q13.2. The expression of gene SCRIB in region 8q24.3 was positively correlated with its copy number (Spearman’s r = 0.522). SCRIB gene is closely related to tumors and is considered to be a key factor of tumor development and metastasis (Zen et al., 2009; Royer and Lu, 2011). It is associated with proliferation, apoptosis, EMT and poor prognosis in CRC (Shen et al., 2021). SCRIB also interacts with the HIPPO signaling pathway in CRC (Enomoto and Igaki, 2011). These results clarified that alterations in specific chromosomal regions may affect the expression of genes, and further influence a series of signaling pathways and biological processes in CRC.

Next, we identified five agents, PD.173,074, Pazopanib, Docetaxel, KU-55933 and Embelin, which may have potential efficacy for high-risk patients in CRC. Of the five agents, PD.173,074 (CMap score = −91.88) and Docetaxel (CMap score = −91.55) were probably the most promising agents. PD-173074 is a fibroblast growth factor receptor (FGFR) inhibitor. The FGFR inhibitors have shown significant antitumor activity against multiple tumor cell lines, including stomach, lung, bladder, endometrium and breast (Rusnati MP and Presta, 2007). Many FGFR inhibitors have been developed to treat various cancers; for example, brivanib (Ayers et al., 2007), AZD4547 (Gavine et al., 2012), E-3810 (Bello et al., 2011), NP603 (Kammasud et al., 2007), etc. Docetaxel is a tubulin polymerization inhibitor. Tubulin polymerization inhibitors inhibit spindle formation by inducing depolymerization of microtubules, block cells in phase M, and eventually induce cell apoptosis (Morris and Fornier, 2008). Therefore, the two drugs are considered to have the most promising therapeutic potential for patients with high risk in CRC.

In addition to the tumorigenic effect, inflammation can also affect the immune system, thereby enhancing the response to chemotherapy (Apetoh et al., 2008). However, inflammation can blunt the beneficial effects of chemotherapy in some cases (Ammirante et al., 2010). Therefore, we explored the relationship between the IRLncSig and immunotherapy. We found high-risk group exhibited higher immune cell infiltration and expression value of immune checkpoints, suggesting that high-risk patients may be more likely to respond to immunotherapy. The submap analysis further confirmed this conclusion, which may provide some help for the development of clinical treatment strategies for patients with CRC.

There are some limitations in this study. Firstly, less clinical information was considered in this study considering the availability of data. It needs to verify the independence of the IRLncSig in a larger cohort containing more clinical information. Secondly, this study conducted a series of bioinformatics analyses based on public databases and may need further biological experimental verification. Finally, this study is a prospective study, and prospective studies may be needed to further examine the prognostic performance of the IRLncSig.

## Conclusion

Collectively, we established a new signature, IRLncSig, based on sixteen inflammation-related lncRNAs. Our analysis suggested that the IRLncSig is an independent and effective prognostic marker and superior to the other four existing prognostic signatures in CRC. Functional enrichment analysis, somatic mutation and copy number alterations were conducted to reveal the biological significance of the IRLncSig. We performed drug sensitivity predictions and identified chemotherapeutic agents that might be effective for high-risk group patients. We also found that high-risk patients were more likely to respond to immunotherapy, especially anti-CTLA4 therapy. These findings could provide some help for the development of clinical treatment strategies in CRC.

## Data Availability

All data were downloaded from public databases. The TCGA-COADREAD cohort (including TCGA-COAD and TCGA-READ) was downloaded from the TCGA database (https://portal.gdc.cancer.gov/). The GSE39582 cohort, GSE38832 cohort and GSE72970 cohort were downloaded from the GEO database (GSE39582: https://www.ncbi.nlm.nih.gov/geo/query/acc.cgi?acc=GSE39582; GSE38832: https://www.ncbi.nlm.nih.gov/geo/query/acc.cgi?acc=GSE38832; GSE72970: https://www.ncbi.nlm.nih.gov/geo/query/acc.cgi?acc=GSE72970). In addition, no new cohorts were generated in this study.
