# Supplementary material for: Identification and validation of an inflammation-related lncRNAs signature for improving outcomes of patients in colorectal cancer
Source: Front Genet. 2022 Sep 30;13:955240. doi: 10.3389/fgene.2022.955240 (PMC9561096; doi:10.3389/fgene.2022.955240)
Supplement: Supplementary file 1 [file DataSheet1.ZIP › Supplementary Materials/Supplementary Figures.docx]

# Identification and validation of an inflammation-related lncRNAs signature for improving outcomes of patients in colorectal cancer

# Supplementary Figures

**
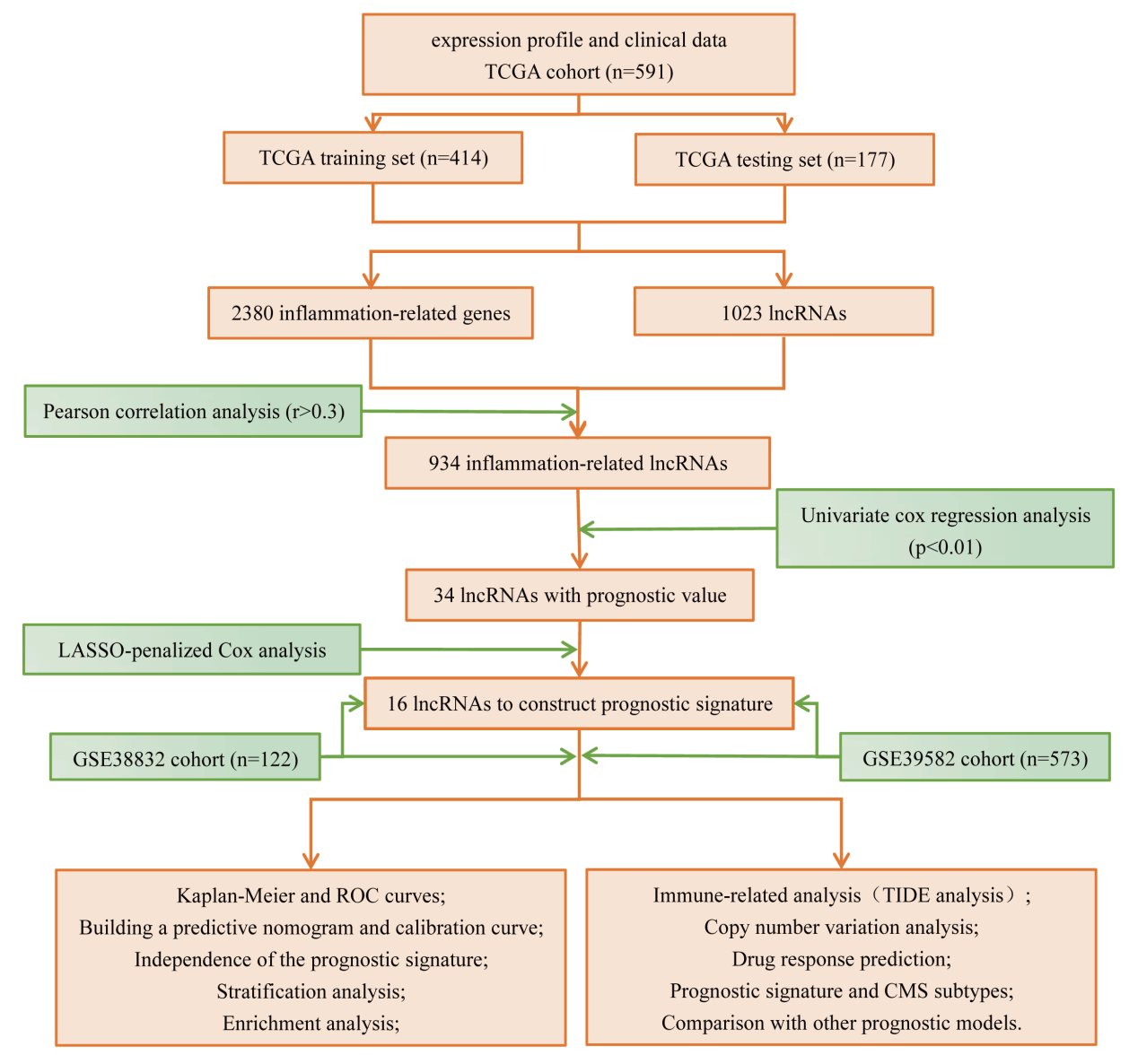
**

### **Figure S1**. Flow charts for the signature construction and analysis in this study.


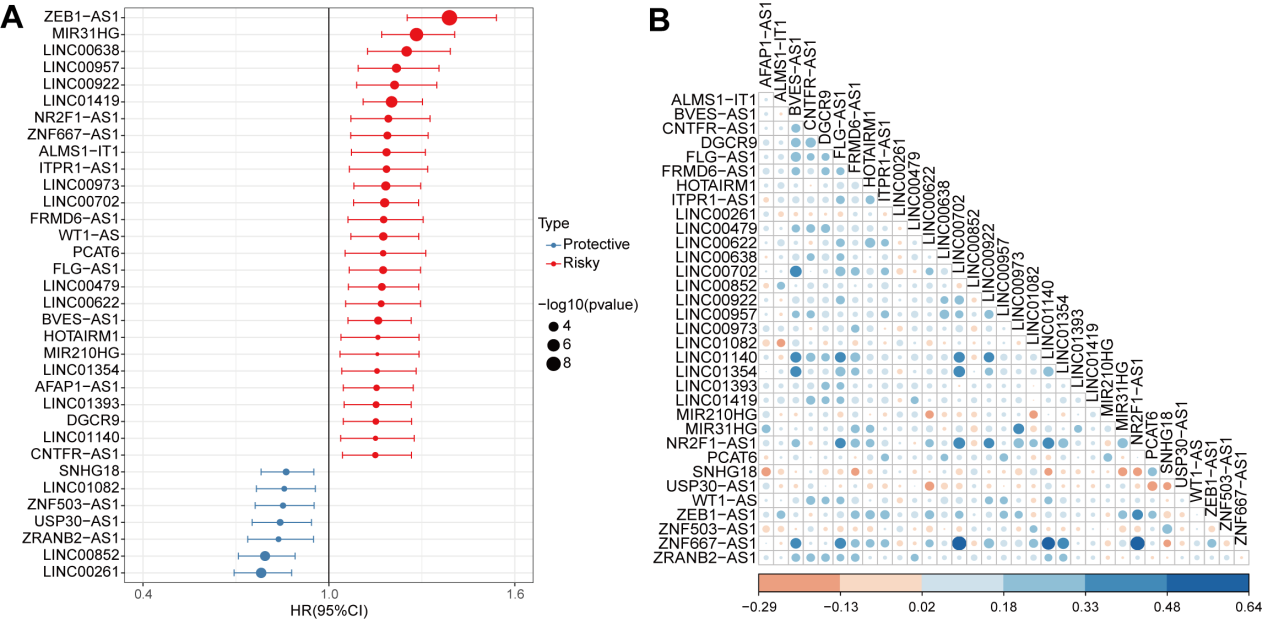


### **Figure S2.** 34 lncRNAs with significant prognostic value. **(A)** Forest plot for 34 lncRNAs identified by univariate Cox regression analysis. **(B)** The co-expression analysis for these lncRNAs through the R package “corrplot”.


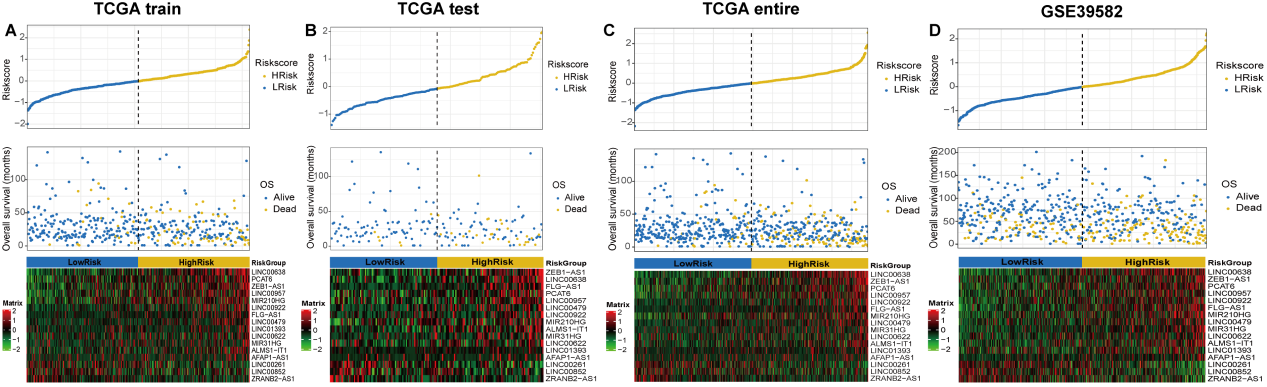


### **Figure S3.** The distribution of risk score and heatmap for the expression level of sixteen lncRNAs in the TCGA training (A), TCGA testing (B), TCGA entire (C) and GSE39582 (D) cohorts.

###
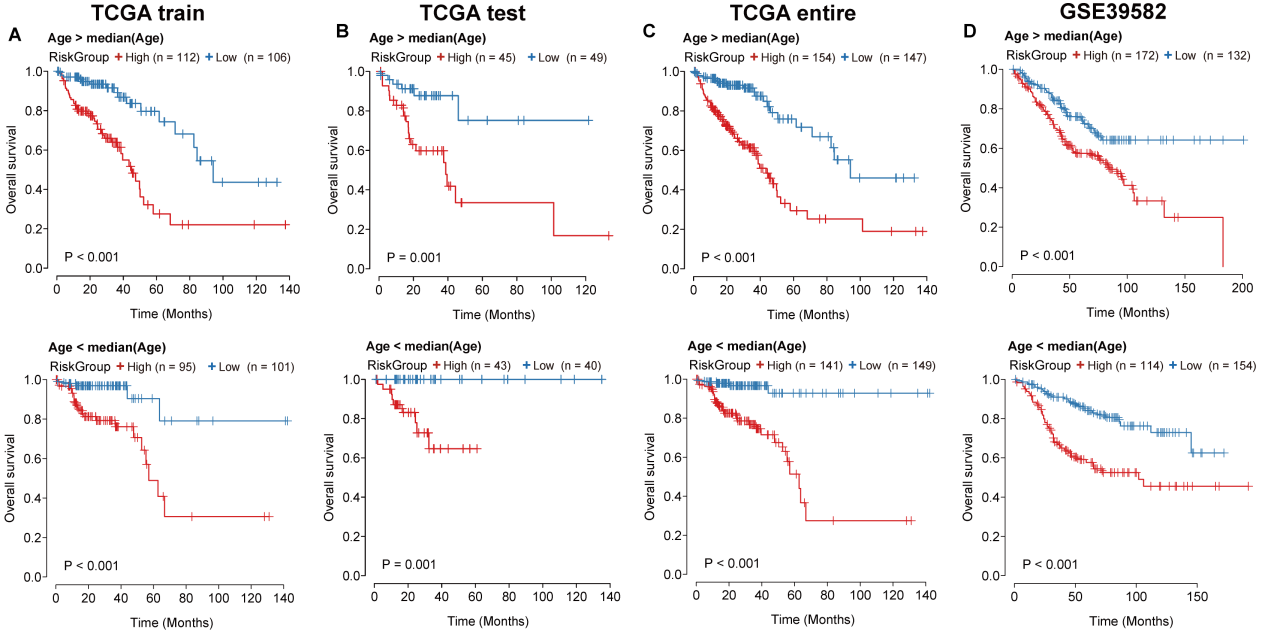
**Figure S4.** Stratification analysis by clinical variable age in the TCGA training (A), TCGA testing (B), TCGA entire (C) and GSE39582 (D) cohorts. The p values were calculated by the Log-rank test.

###
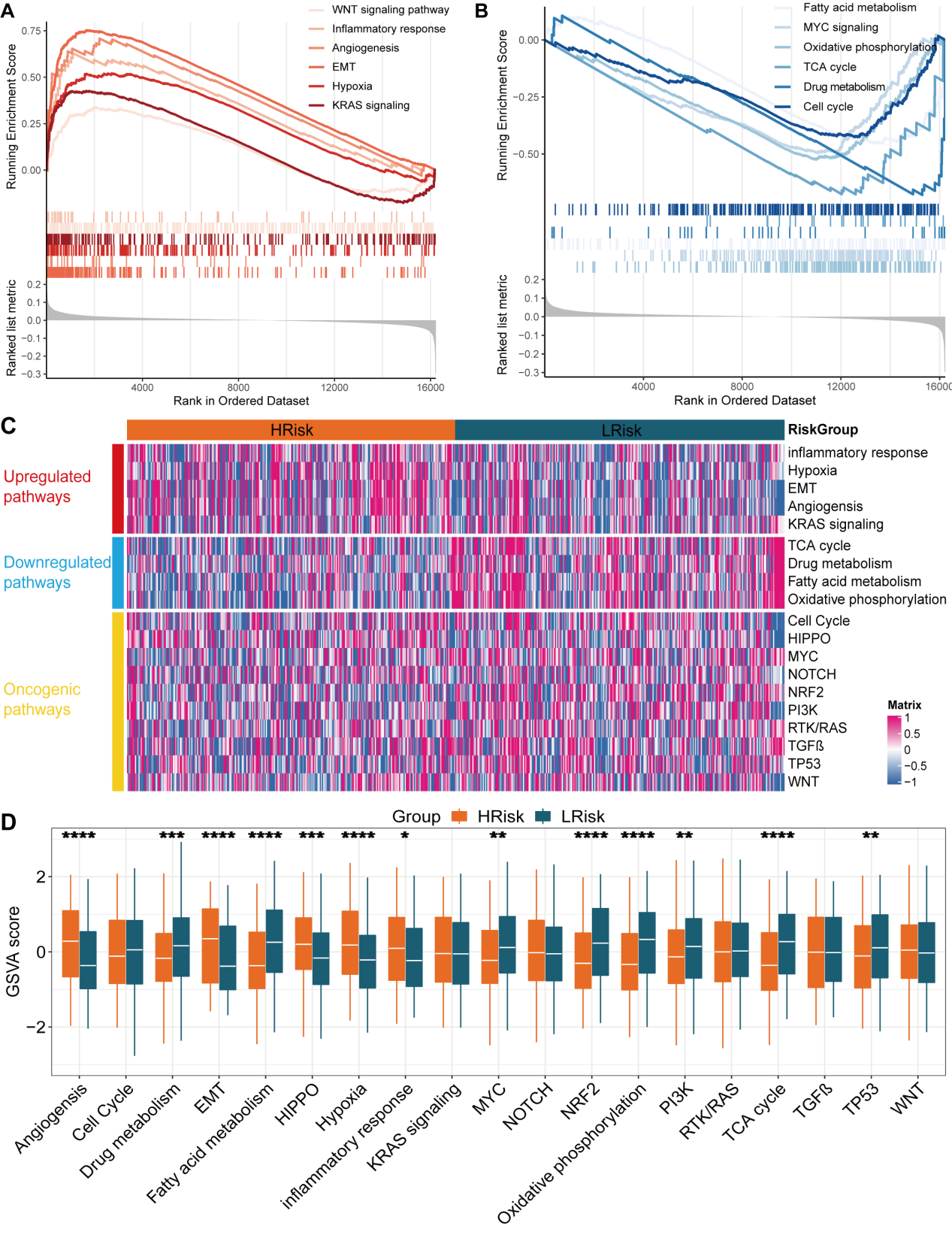
**Figure S5.** Function enrichment analysis in the GSE39582 cohort. **(A-B)** Gene set enrichment analysis based on GO, KEGG, Hallmark and Reactome. **(C)** Heatmap for the GSVA score of upregulated, downregulated and oncogenic pathways. **(D)** Boxplot for the GSVA score of upregulated, downregulated and oncogenic pathways between two subgroups. The p values were calculated by Wilcoxon test (*p < 0.05; **p < 0.01; ***p < 0. 001; ****p < 0. 0001).

### **
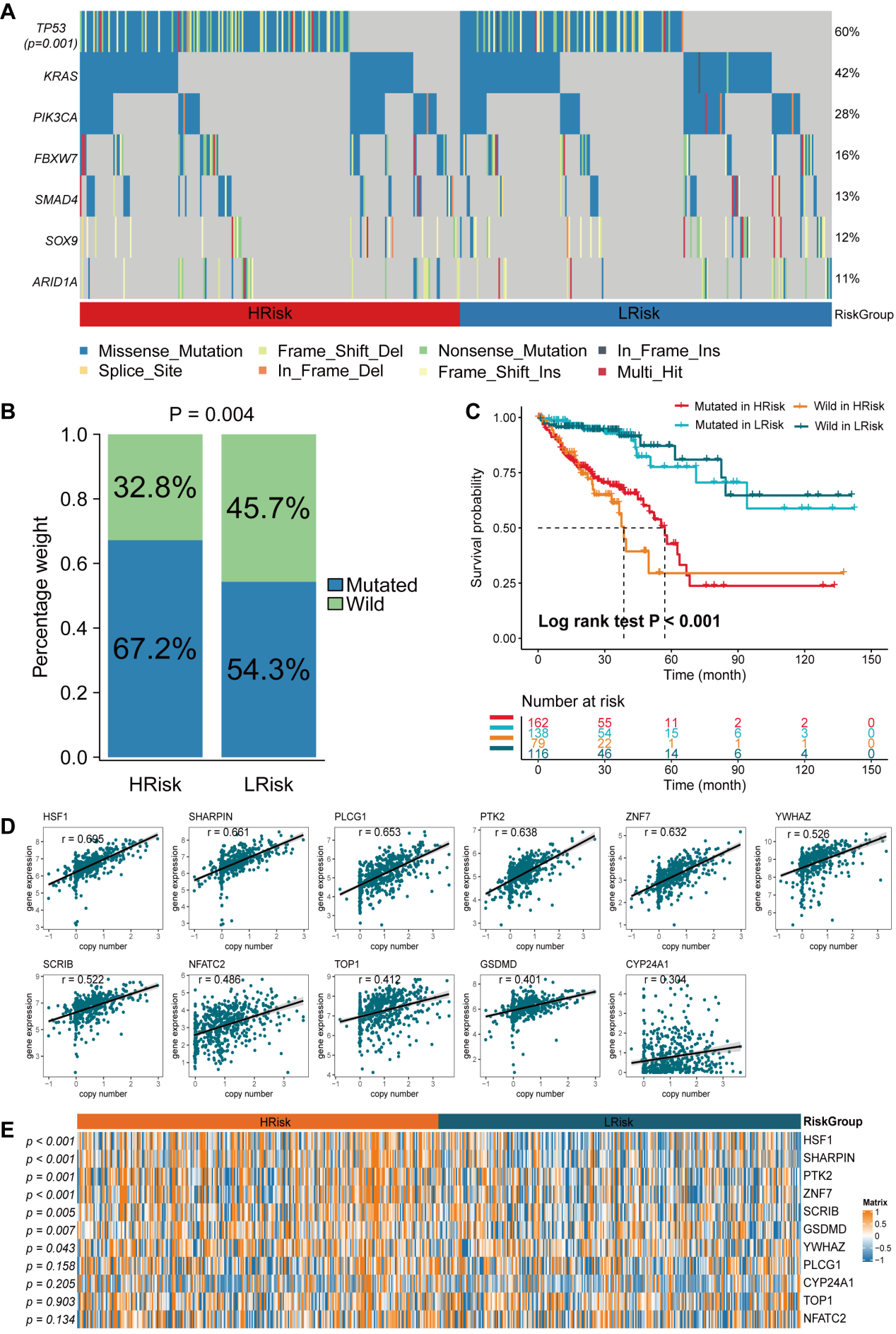
Figure S6.** Characterization of somatic mutation and copy number variation in the TCGA cohort. **(A)** Oncoplot for the top 7 drivers genes identified by MutSigCV. **(B)** Barplot for the distribution of TP53 mutation between two risk groups. The p values were calculated by Fisher’s exact test. **(C)** Kaplan-Meier curve for four subgroups based on TP53 mutation status and risk group. The p values were calculated by the Log-rank test. **(D)** Correlation between copy number and expression value of 11 inflammatory genes. **(E)** Heatmap for the expression level of eleven inflammatory genes. The p values were calculated by the Wilcoxon test.

###
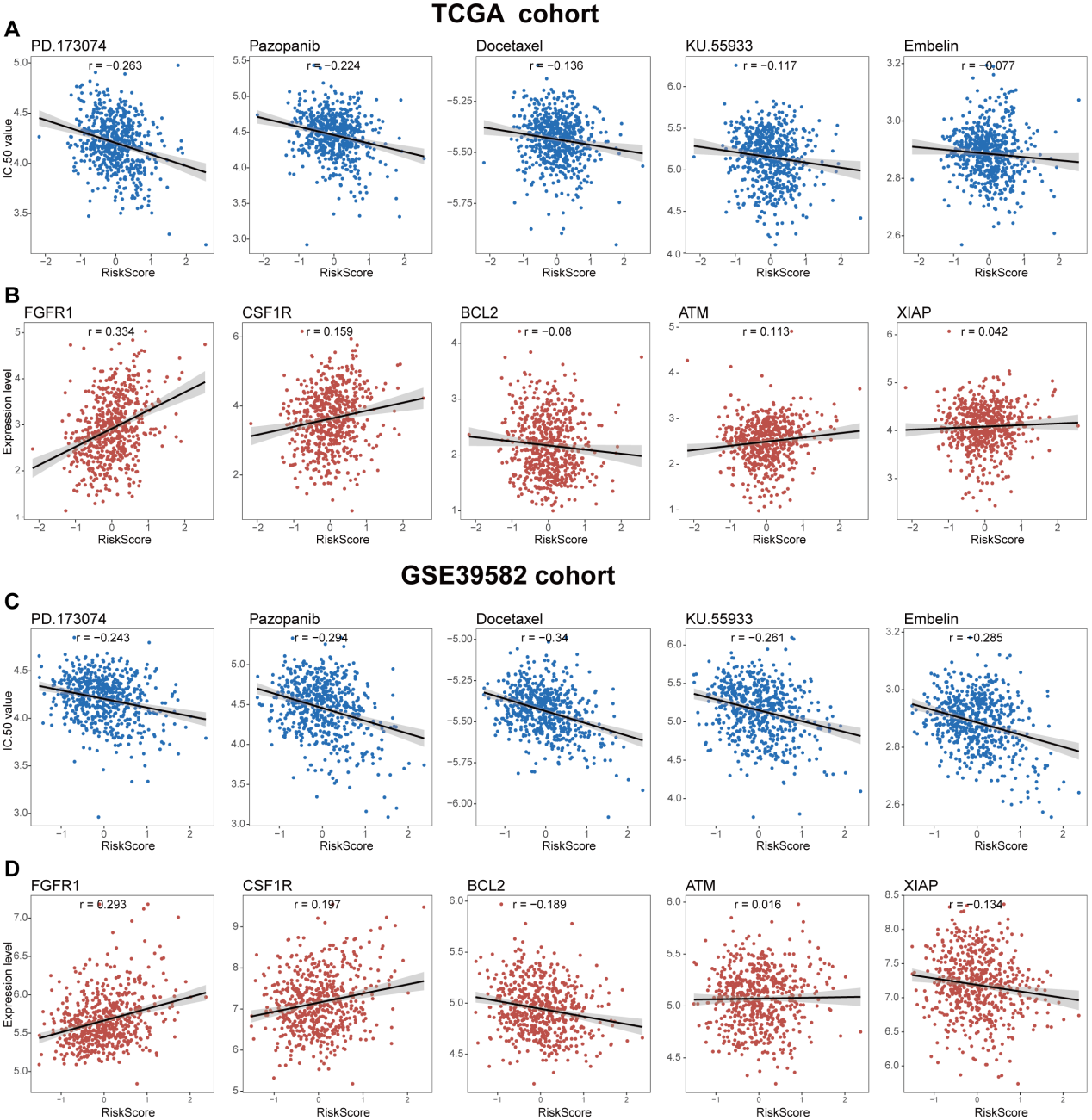
**Figure S7.** The Spearman’s correlation analysis of the IC50 value and target expression level of five drugs with the risk score. **(A, C)** IC50 value in the TCGA (A) and GSE39582 (C) cohorts. **(B, D)** Drug target expression levels in the TCGA (B) and GSE39582 (D) cohorts.

**
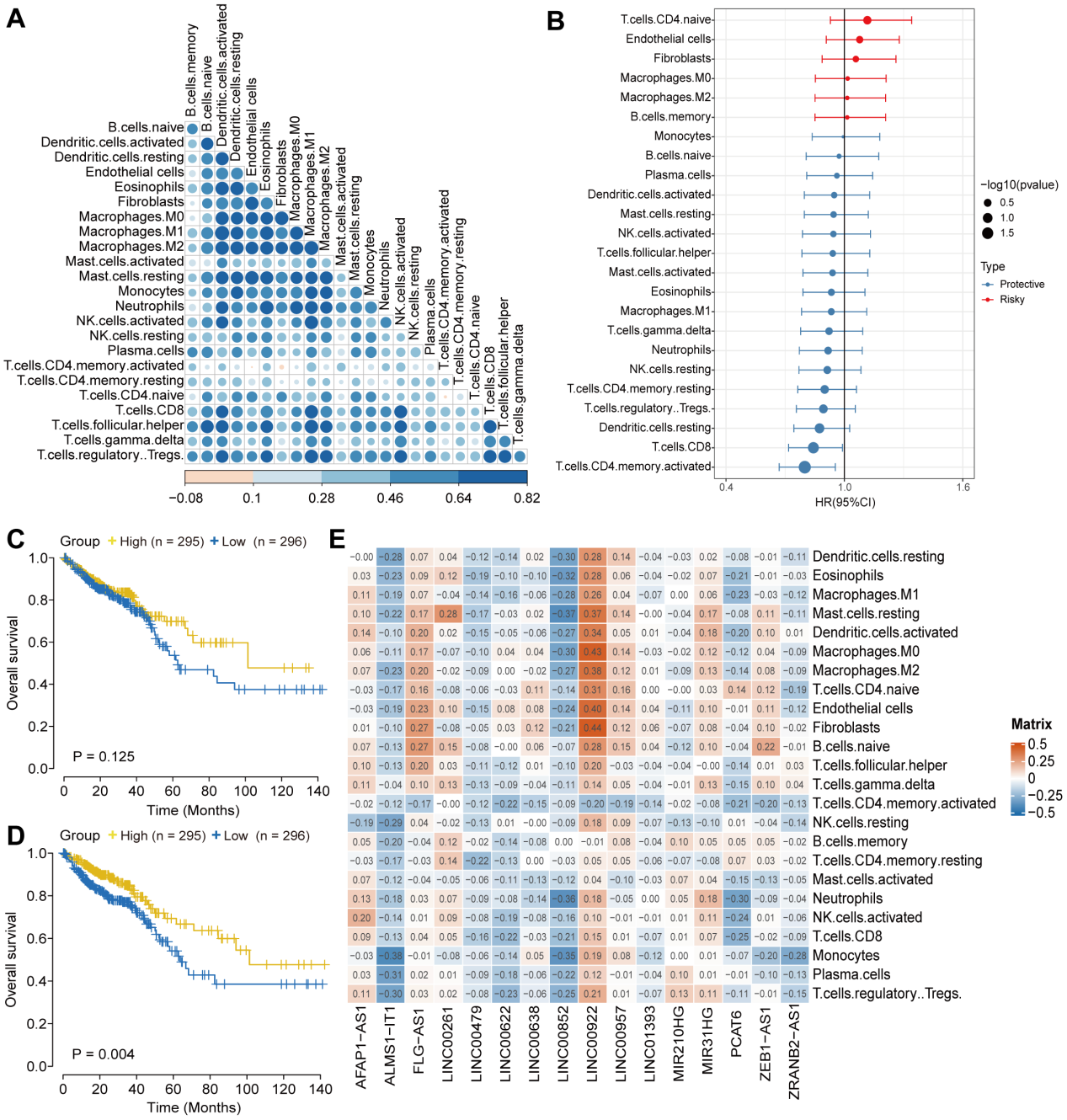
**

### **Figure S8.** Immune cells in the tumor microenvironment. **(A)** The interplay of 22 immune cells and 2 stromal cells. **(B)** Forest plot for the prognostic value of 22 immune cells and 2 stromal cells. **(C-D)** Survival analysis is based on the infiltration level of two immune cells, CD8 T cells (C) and activated memory CD4 T cells (D). The p values were calculated by the Log-rank test. **(E)** Heatmap for the correlation between 16 inflammation-associated lncRNAs and immune cells.


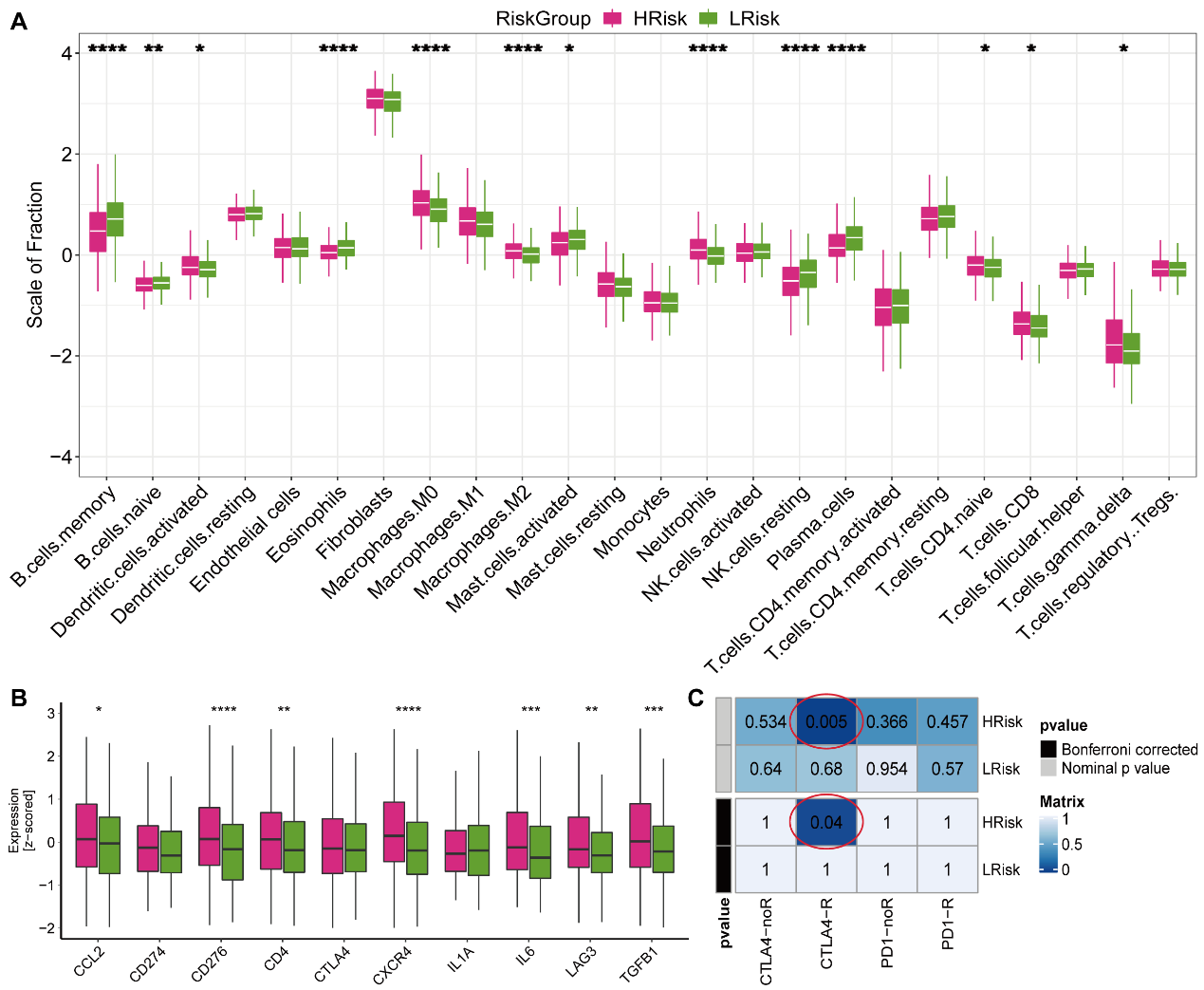


### **Figure S9.** Immune infiltration and the response to immunotherapy in the GSE39582 cohort. **(A)** Boxplot for the abundance of 22 immune cells and 2 stromal cells estimated by ssGSEA. The p values were calculated by Wilcoxon test (*p < 0.05; **p < 0.01; ***p < 0. 001; ****p < 0. 0001). **(B)** Boxplot for the expression level of 10 immune checkpoints. The p values were calculated by Wilcoxon test (*p < 0.05; **p < 0.01; ***p < 0. 001; ****p < 0. 0001). **(C)** Submap analysis for predicting the response to immunotherapy.

**
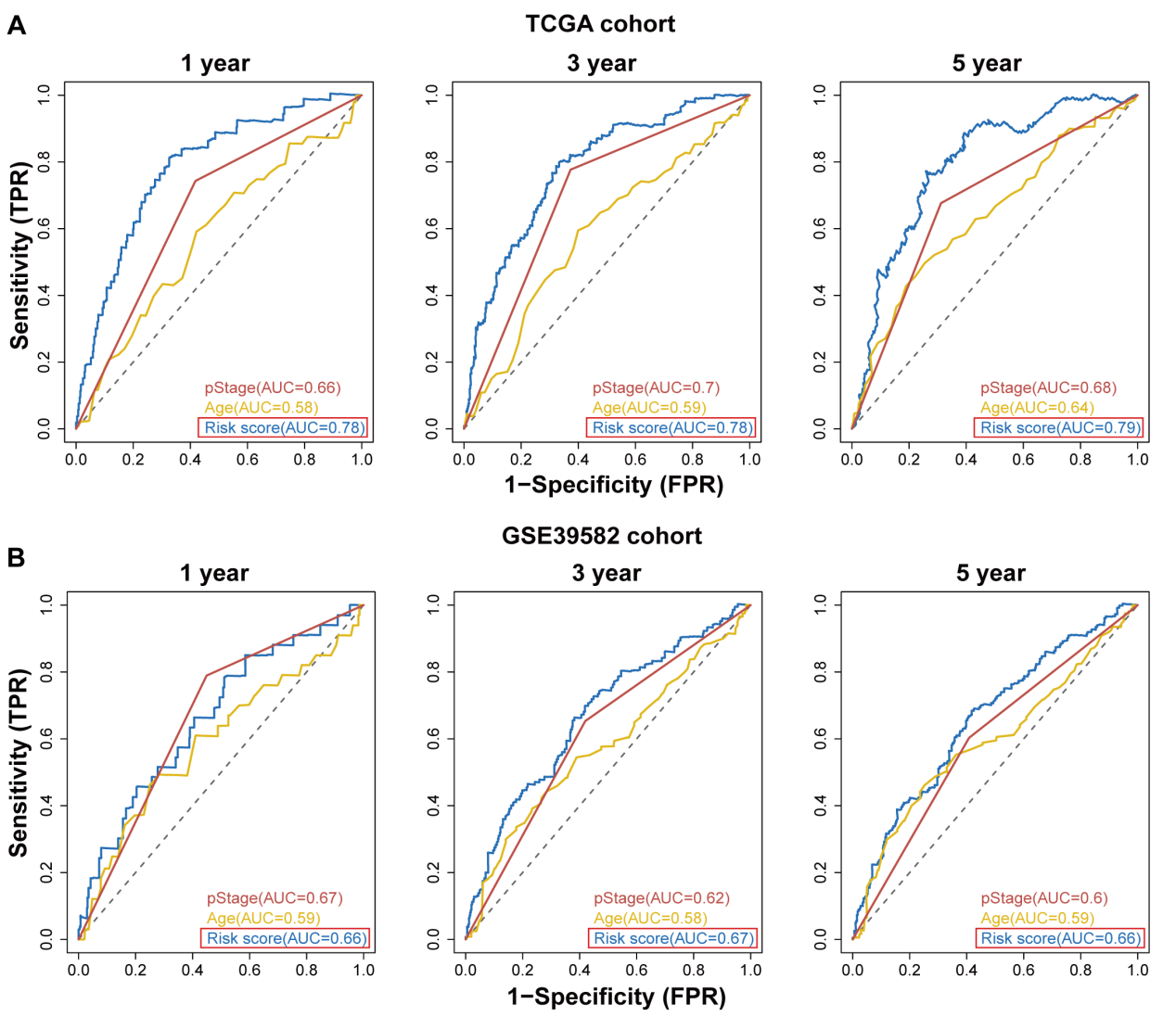
**

### **Figure S10.** Comparison of ROC curves among IRLncSig and other clinical factors, including age and pathologic stage in the TCGA entire (A) and GSE39582 (B) cohorts.


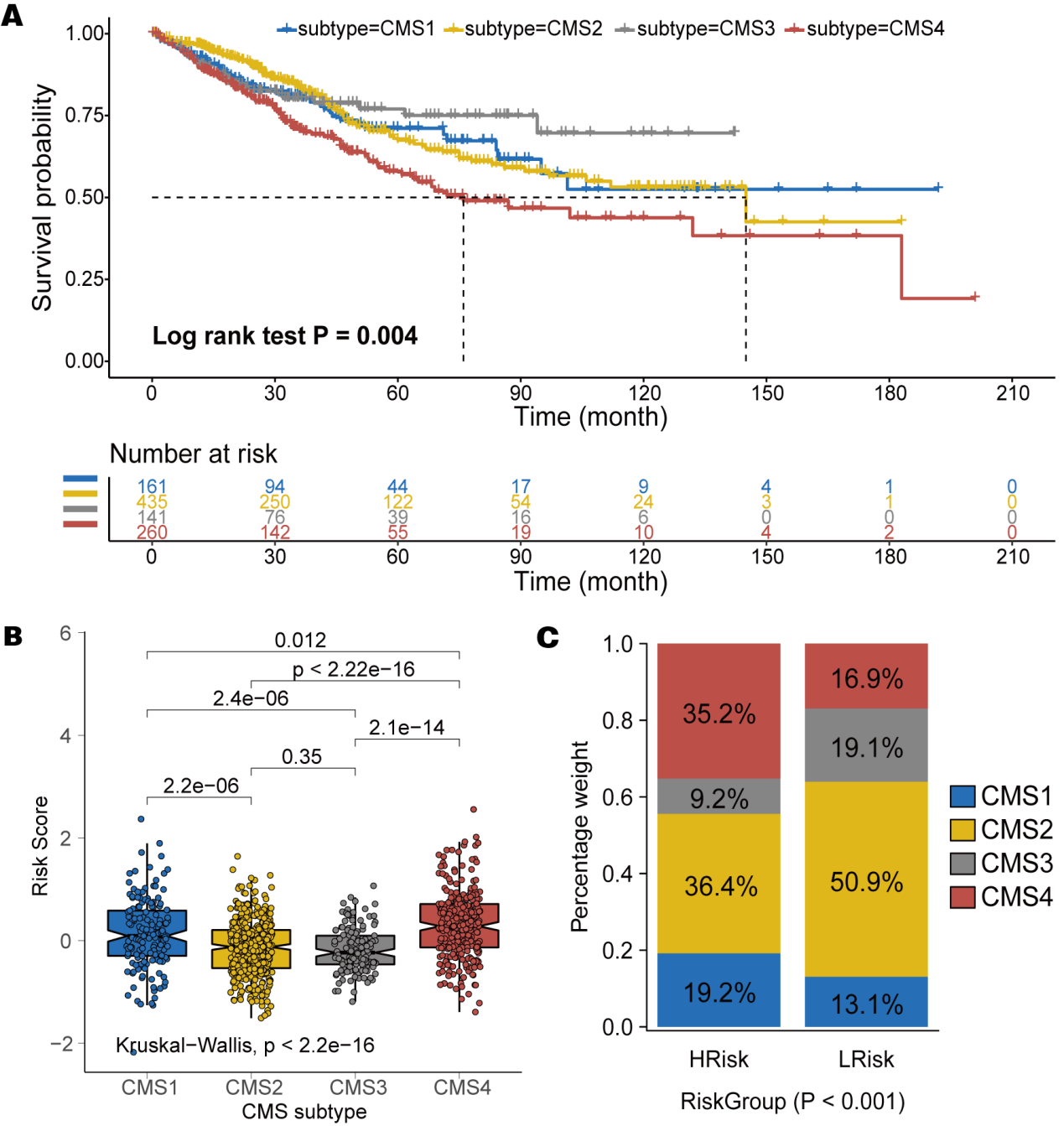


### **Figure S11.** The association between the IRLncSig and CMS subtypes in CRC. **(A)** Kaplan–Meier curves for four CMS subtypes. The p values were calculated by the Log-rank test. **(B)** The difference of the risk score in the four CMS subtypes. The p values were calculated by the Kruskal–Wallis test. **(C)** Bar plots showing the distribution of four CMS subtypes in different risk groups. The p values were calculated by the Chi-square test.


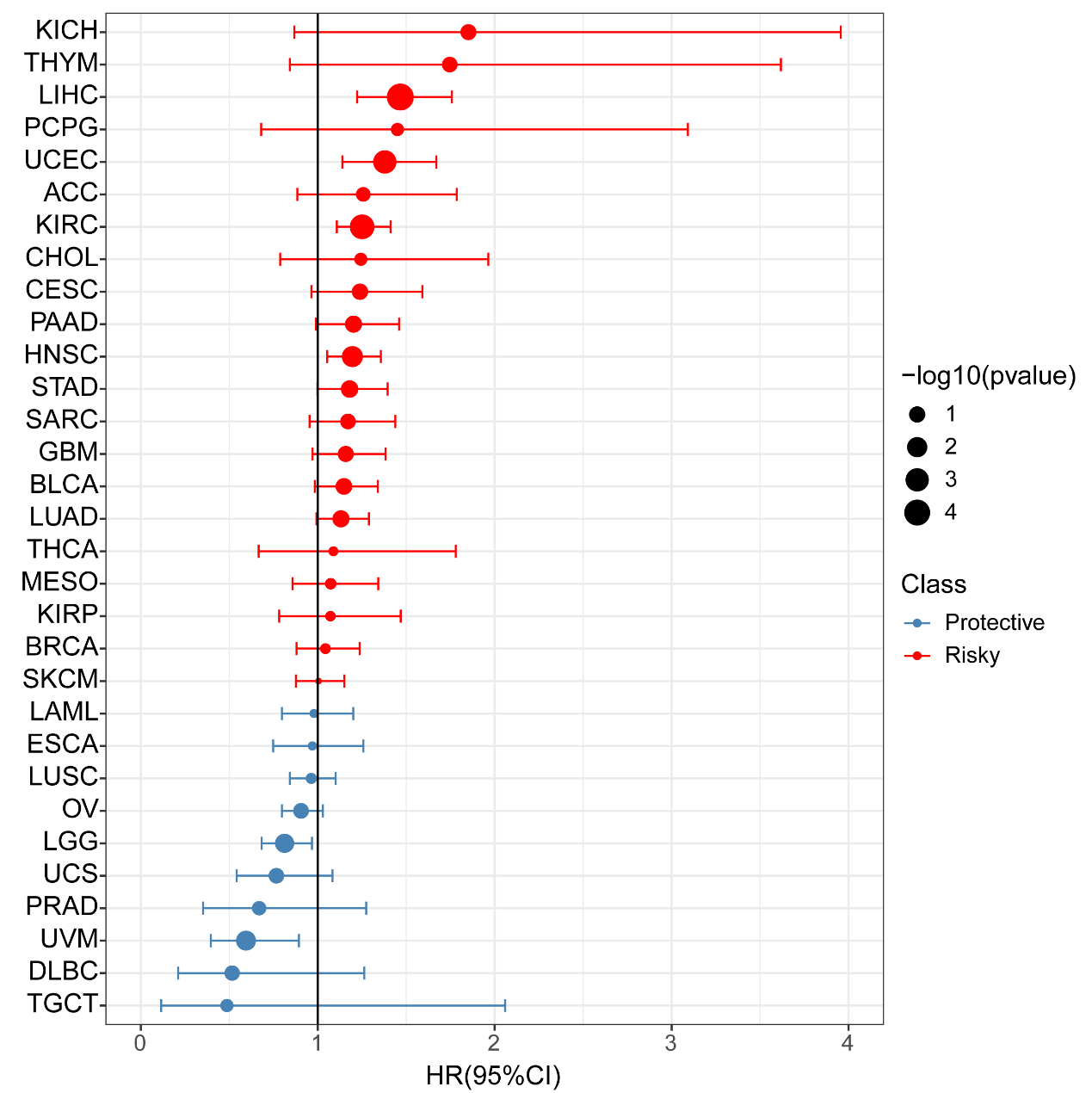


### **Figure S12.** The prognostic value of the IRLncSig in pan-cancer analysis.
